# A Novel Glucocerebrosidase Allosteric Activator Prevents MPTP-Induced Neurodegeneration in Mice

**DOI:** 10.3390/ijms27219602

**Published:** 2026-10-27

**Authors:** Anton Emelyanov, Alena Kopytova, Anna Lavrinova, Georgy Rychkov, Margarita Rudenok, Ivan Rybolovlev, Farid Ibatullin, Victoria Pidyurchina, Alexandra Artynyuk, Mikhail Nikolaev, Anastasia Bezrukova, Alexander Stepakov, Vitali Boitsov, Galina Baydakova, Elena Grigor’eva, Sophia Pavlova, Suzanna Partevian, Ekaterina Semenova, Irina Miliukhina, Maxim Nesterov, Denis Abaimov, Vadim Bezrukikh, Alla Timofeeva, Varvara Kvanchiani, Ekaterina Sergeeva, Maria Shadrina, Tatiana Usenko, Ekaterina Zakharova, Petr Slominsky, Sofya Pchelina

**Affiliations:** 1 Petersburg Nuclear Physics Institute Named by B.P. Konstantinov of National Research Center «Kurchatov Institute», 188300 Gatchina, Russia; 2 Department of Molecular Genetic and Nanobiological Technologies, Scientific Research Center, Pavlov First Saint-Petersburg State Medical University, 197022 Saint-Petersburg, Russia; 3 Higher School of Biomedical Systems and Technologies, Institute of Biomedical Systems and Biotechnology, Peter the Great Saint-Petersburg Polytechnic University, 195251 Saint-Petersburg, Russia; 4 National Research Center «Kurchatov Institute», 123182 Moscow, Russia; 5 Department of Organic Chemistry, Saint Petersburg State Institute of Technology (Technical University), 190013 Saint-Petersburg, Russia; 6 Laboratory of Nanobiotechnologies, Alferov University, 194021 Saint-Petersburg, Russia; 7 Research Centre for Medical Genetics, 115522 Moscow, Russia; 8 Institute of Cytology and Genetics Siberian Branch of Russian Academy of Sciences, 630090 Novosibirsk, Russia; 9 N.P. Bechtereva Institute of the Human Brain of Russian Academy of Sciences, 197376 Saint-Petersburg, Russia; 10 Scientific Center of Biomedical Technologies, Federal Medical and Biological Agency of Russia, 143442 Moscow, Russia; 11 Research Centre of Neurology, 125367 Moscow, Russia; 12 Almazov National Medical Research Centre, 197341 Saint-Petersburg, Russia; 13 Department of Neurology, Pavlov First Saint-Petersburg State Medical University, 197022 Saint-Petersburg, Russia

**Keywords:** glucocerebrosidase activator, Parkinson’s disease, Gaucher disease, *GBA1* variants, glucocerebrosidase, allosteric binding site, PBMC-derived macrophages, iPSC-derived dopaminergic neurons, MPTP-induced mouse model of Parkinson’s disease

## Abstract

Mutations in the *GBA1* gene, encoding lysosomal β-glucocerebrosidase (GCase), cause Gaucher disease (GD) and represent the most common genetic risk factor for Parkinson’s disease (PD). To target this pathway, therapeutic approaches for both GD and PD are actively being developed, focusing on two main drug classes: pharmacological chaperones (e.g., ambroxol) and allosteric GCase activators. Here, we characterize a novel allosteric GCase activator, N2, a derivative of the non-inhibitory chaperone NCGC607, and evaluate its therapeutic potential using computational, in vitro, and in vivo approaches. Molecular docking and molecular dynamics simulations indicated that N2 binds within the allosteric site of the mutant p.N370S GCase structure. In patient-derived cells (peripheral blood mononuclear cell-derived macrophages and induced pluripotent stem cell-derived dopaminergic neurons from GD and GBA1-PD patients), N2 enhanced GCase lysosomal translocation, modulated its enzymatic activity and protein level, and reduced substrate accumulation. Furthermore, we demonstrate for the first time that N2 exerts robust neuroprotective effects in an in vivo 1-methyl-4-phenyl-1,2,3,6-tetrahydropyridine (MPTP)-induced mouse model of PD. Specifically, N2 treatment attenuated MPTP-induced hexosylsphingosine accumulation in the prefrontal cortex, and ameliorated both tyrosine hydroxylase-positive neuronal loss in the substantia nigra and dopamine depletion in the striatum. Our findings first demonstrated the neuroprotective properties of a GCase allosteric activator on an MPTP-induced mouse model of PD and supported the emerging perspectives of such compounds for the treatment of GCase-related disorders.

## 1. Introduction

The *GBA1* gene encodes for the lysosomal enzyme β-glucocerebrosidase (GCase). Biallelic mutations in the *GBA1* gene lead to dysfunction of the GCase enzyme, resulting in accumulation of its substrate glucosylceramide (GlcCer) in cells, lysosomal dysfunction and the development of Gaucher disease (GD), a rare autosomal recessive disorder related to lysosomal storage disorders. As a lysosomal hydrolase, GCase is synthesized in the endoplasmic reticulum (ER) and is transported through the Golgi apparatus to the lysosomes, where it is essential for the conversion of GlcCer into glucose and ceramide [1,2,3]. The most common *GBA1* pathogenic variants (p.L444P, p.N370S) associated with PD affect the polypeptide chain’s ability to fold into its native structure, thereby compromising its capacity to reach lysosomes [4,5]. Clinical manifestations of the most common form of GD (nonneuropathic, type 1) include enlargement of the spleen and liver, platelet deficiency, anemia and bone disease. Types 2 and 3 of GD are neuronopathic forms [6,7].

*GBA1* variants are the major genetic risk factor for the second most common neurodegenerative disorder, Parkinson’s Disease (PD) (5–20% of PD cases), increasing PD risk by up to 10 times [2,5,8,9,10,11]. To date, it has become obvious that distinct clinical features, biological signatures, and rates of progression suggest the existence of multiple subtypes of PD, among which PD associated with *GBA1* pathogenic variants (GBA1-PD) is now recognized as a separate entity within the spectrum of parkinsonism [11,12]. GBA1-PD is characterized by an earlier onset of disease, faster motor and cognitive progression, and a higher rate of non-motor symptoms [12,13,14].

We and others have demonstrated that GBA1-PD patients exhibit the decreased GCase activity and an accumulation of the substrate GlcCer and other downstream metabolites, including the deacetylated form of GlcCer glucosylsphingosine (GlcSph), also known as LysoGb1, in blood [11,15,16,17,18,19,20] and patient-derived cells [20,21,22] along with alpha-synuclein accumulation—a key protein in the pathogenesis of PD, autophagy disturbances and inflammation [11,23,24,25,26,27,28]. However, a full understanding of the molecular basis of GBA1-PD pathogenesis is absent. A toxic “gain-of-function” mechanism as the consequence of *GBA1* mutations suggests ER stress and proteostatic imbalance due to GCase misfolding [29].

In recent years, the increasing evidence, including our own data, suggests that a decrease in GCase activity could be relevant to the pathogenesis of idiopathic PD (iPD) as well [30,31,32,33,34]. The bidirectional loop was suggested by in vitro studies indicating that selective loss of GCase activity leads to alpha-synuclein accumulation that in its turn could inhibit GCase activity [4,35]. However, the role of disturbances in sphingolipid metabolism in iPD remains contradictory [36,37].

Common therapeutic approaches for the treatment of GD include enzyme replacement therapy, which involves intravenous injections of recombinant GCase, and substrate reduction therapy, the oral use of small molecule inhibitors to target GlcCer synthase and decrease the production of GlcCer. While enzyme replacement therapy is effective for GD type 1, it is not applicable for types 2 and 3, as well as for GBA1-PD, because the recombinant enzyme cannot penetrate the blood–brain barrier. Substrate reduction therapy with the GlcCer inhibitor venglustat demonstrated no benefit in GBA1-PD patients [38].

An alternative approach to the treatment of GBA1-PD, as well as neuronopathic forms of GD, that is now widely discussed involves the use of pharmacological chaperones (PCs) or GCase activators—small chemical compounds that bind to the GCase enzyme and enhance its trafficking to lysosomes [39].

To date, two distinct classes of GCase-targeted PCs have been described: inhibitory and non-inhibitory (allosteric) chaperones [7].

One of the most promising GCase PCs with unique chaperone characteristics is a well-known mucolytic agent ambroxol (ABX), which has been shown to effectively restore GCase function [40,41,42,43,44,45] and reduce levels of both total and phosphorylated (Ser 129) alpha-synuclein protein in both in cell and animal models [41,43,46,47,48], including experiments conducted on non-human primates [49].

Although ABX has shown efficacy in cell-based and animal models, as well as in clinical trials, allosteric modulators of GCase are believed have more benefits due to their selective action on GCase [40,41,42,43,44,45,49]. Promising GCase non-inhibitory PCs NCGC00241607 (NCGC607) and NCGC00188758 (NCGC758) have been uncovered and investigated in vitro for their potential in treating GD and PD [50].

Nowadays their chemical derivatives have been suggested as potential GCase activators [51,52].

To our knowledge, three allosteric GCase activators (LTI291 (BIA 28–6156)—analog of NCGC758, GT-02287 and VQ-101) have already been included in clinical trials [29,53,54]. However, no studies have reported on the neuroprotective effects of allosteric GCase activators in animal models yet.

**Figure 1:**
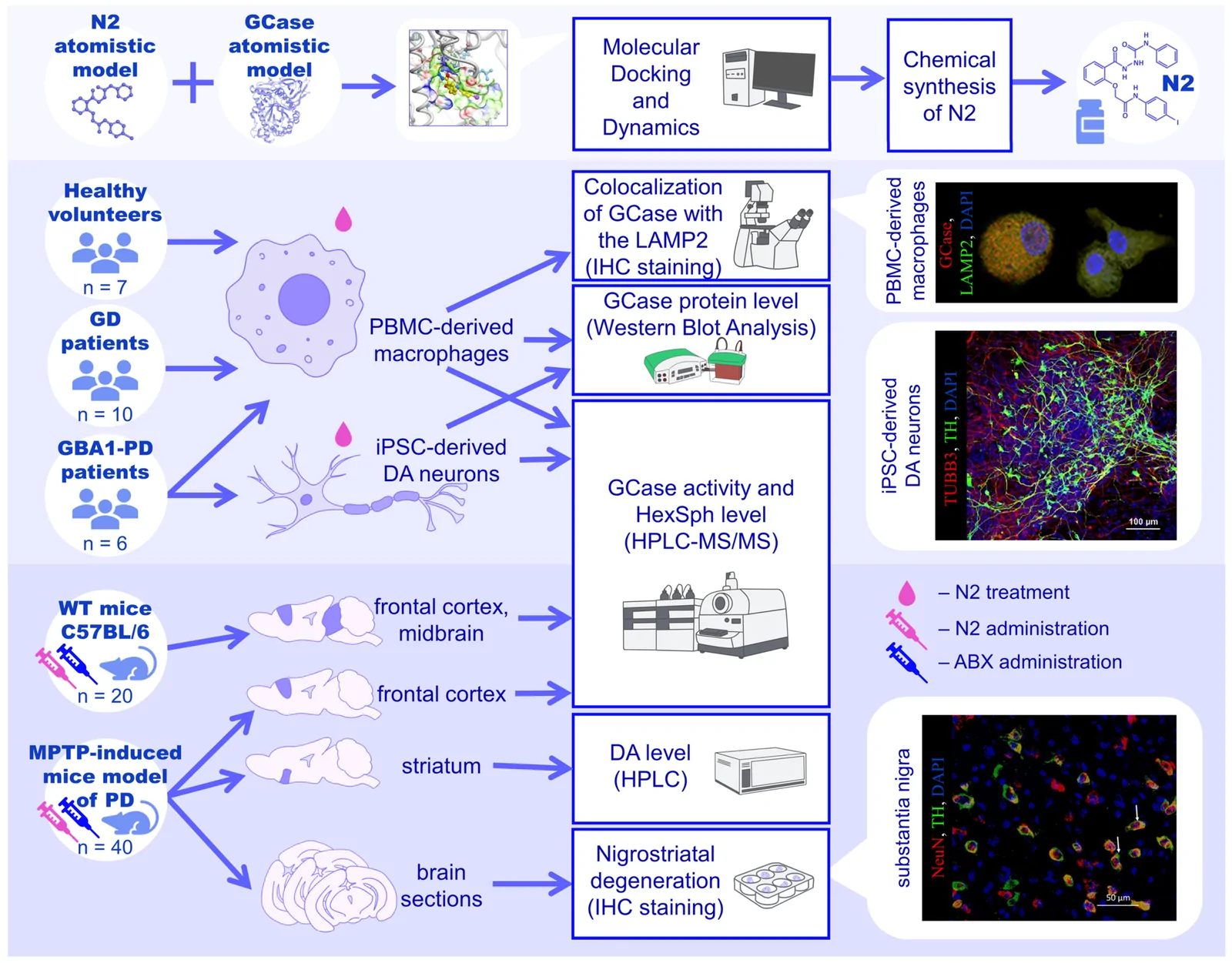
Scheme of experiment. ABX, ambroxol; DA, dopaminergic; GBA1-PD, Parkinson’s disease associated with *GBA1* pathogenic variants; GCase, β-glucocerebrosidase; GD, Gaucher disease; HPLC, high-performance liquid chromatography; HPLC-MS/MS, high-performance liquid chromatography coupled with tandem mass spectrometry; IHC, immunohistochemical; iPSC, induced pluripotent stem cell; MPTP, 1-methyl-4-phenyl-1,2,3,6-tetrahydropyridine; PBMC, peripheral blood mononuclear cell; PD, Parkinson’s disease; WT, wild-type. The schematic was drawn using GoPaint v12.4.2.120 (Huawei, China).

In this study, we characterized the novel GCase activator N2 (an analog of NCGC607), assessing its binding energy in silico and its ability to rescue GCase activity and expression in patient-derived cells (peripheral blood mononuclear cell (PBMC)-derived macrophages and induced pluripotent stem cell (iPSC)-derived dopaminergic (DA) neurons), and its neuroprotective efficacy in an 1-methyl-4-phenyl-1,2,3,6-tetrahydropyridine (MPTP)-induced mouse model of parkinsonism (Figure 1).

## 2. Results

### 2.1. Computational Prediction of N2 Binding in the BS1 Allosteric Pocket of p.N370S GCase

To investigate a potential structural basis for the GCase-modulating effects of compound N2 (2-(2-(4-iodophenylamino)-2-oxoethoxy)benzoyl-N-phenylhydrazine carboxamide), we performed molecular docking and molecular dynamics (MD) simulations using the previously identified BS1 allosteric pocket of p.N370S GCase [43,55]. BS1 is located in Domain 2, directly beneath Loop 6 (residues 311–319), adjacent to but not overlapping the substrate-binding cleft. The pocket is delimited by Loop 6, Loop 7 (residues 345–349), and α-helix 7 (residues 357–371), and is partially formed by a cavity generated by the p.N370S substitution. Thus, BS1 lies in close spatial proximity to the catalytic region, including the vicinity of the catalytic nucleophile Glu340, while remaining outside the active-site cavity.

Five distinct docking poses of N2 (designated as p3, p4, p5, p10, and p15) were selected as starting configurations for 50 ns explicit-solvent MD simulations to verify their thermodynamic and structural stability.

The stability of each complex was assessed by monitoring ligand retention in the binding pocket and by calculating the binding free energy (ΔG_bind_) using the MM/GBSA approach (Table 1). During the simulations, complexes initiated from poses p3, p4, and p5 were unstable, whereas poses p10 and p15 retained N2 within BS1 throughout the 50 ns trajectories. Among these computationally stable poses, p10 showed the most favorable MM/GBSA binding free-energy estimate (−46.69 ± 7.95 kcal/mol), while p15 showed a less favorable estimate (−31.09 ± 4.60 kcal/mol) (Table 1). Therefore, p10 was selected as the representative predicted BS1 binding mode for subsequent structural analysis. These MM/GBSA values should be interpreted for relative ranking of the simulated poses and not as experimentally determined binding affinities.

**Figure 2:**
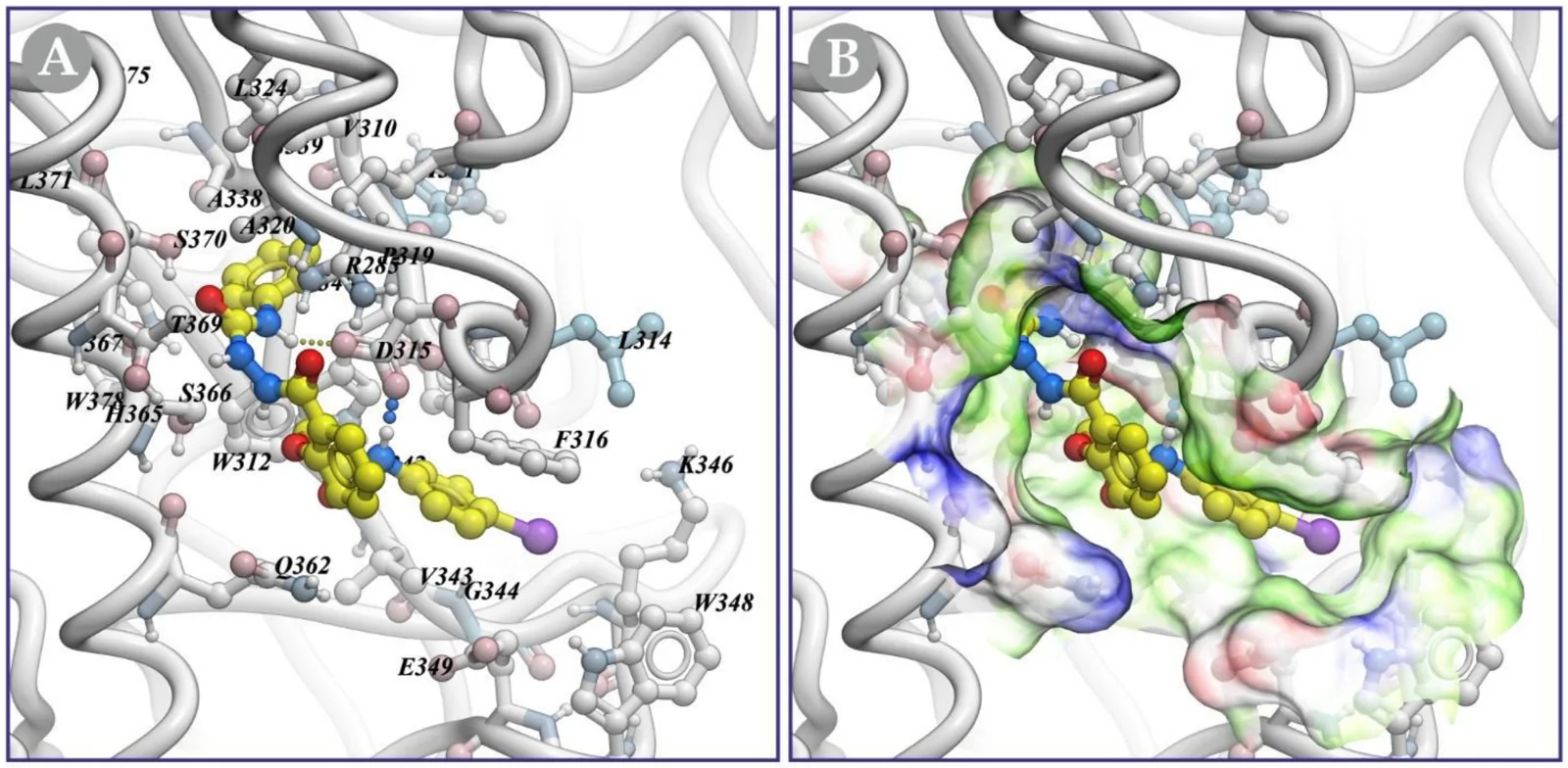
Computationally predicted binding mode of compound N2 in the allosteric site BS1 of the p.N370S mutant GCase. Representative median conformation from the most populated MD cluster for pose p10 (**A**). Compound N2 is shown as a yellow ball-and-stick model with nitrogen in blue, oxygen in red, and iodine in purple. Amino acid residues within a 5.0 Å radius of the ligand are shown and labeled. Intermolecular hydrogen bonds are shown as dashed lines. Molecular surface representation of the BS1 binding pocket occupied by compound N2, illustrating the steric complementarity and accommodation of the activator within the allosteric cavity (**B**).

Cluster analysis of the MD trajectory for the most stable complex (p10) revealed the representative median conformation of N2, in which it occupies the elongated BS1 cavity beneath Loop 6 (Figure 2). In this binding mode, compound N2 adopts an extended conformation that complements the topology of the allosteric cavity formed in the vicinity of the p.N370S substitution (Figure 2B). Thus, N2 is not predicted to occupy the catalytic site directly.

In the p10 model, N2 occupies the elongated BS1 cavity beneath Loop 6. The 4-iodophenyl group is oriented toward the subpocket formed by Val343, Gly344, Lys346, Trp348, Glu349, and Phe316, whereas the terminal phenylhydrazine carboxamide moiety extends toward the region adjacent to Ser370. The predicted interaction network includes contacts with residues from Loop 6 and the adjacent α-helix 7, including Arg285, Trp312, Asp315, Phe316, Ala338, Ser366, Thr369, Ser370, Val375, and Trp378 (Figure 2). The central aromatic core and amide linkers make contacts with Trp312, Asp315, Phe316, Gln362, and His365. A network of intermolecular hydrogen bonds (notably involving Arg285, Asp315, and Ser370) stabilizes the orientation of N2, anchoring the activator in the BS1 site and mediating conformational stabilization of the adjacent loops in the p.N370S mutant enzyme.

### 2.2. The Effects of N2 on Patient-Derived Cells (PBMC-Derived Macrophages and iPSC-Derived DA Neurons) and MPTP-Induced Mouse Model of PD

To evaluate the effects of the novel allosteric GCase activator N2, we performed a two-stage study combining complementary in vitro and in vivo approaches. First, we assessed the effects of N2 on patient-derived cells, including PBMC-derived macrophages and iPSC-derived DA neurons, focusing on GCase activity, GCase protein abundance, its translocation to lysosomes, and hexosylsphingosine (HexSph) level (galactosylsphingosine (GalSph) and GlcSph). Then we evaluated whether the effects observed in patient-derived cells translated into biochemical and neuroprotective effects in vivo using wild-type mice and an MPTP-induced mouse model of parkinsonism.

#### 2.2.1. The Effects of N2 Treatment on PBMC-Derived Macrophages from GD, GBA1-PD and Controls

In this study, we used PBMC-derived macrophages from patients with GD and GBA1-PD to evaluate the effects of the novel GCase allosteric activator N2 on GCase activity and level of GCase, as well as the degree of GCase translocation into lysosomes and the substrate level.

**Figure 3:**
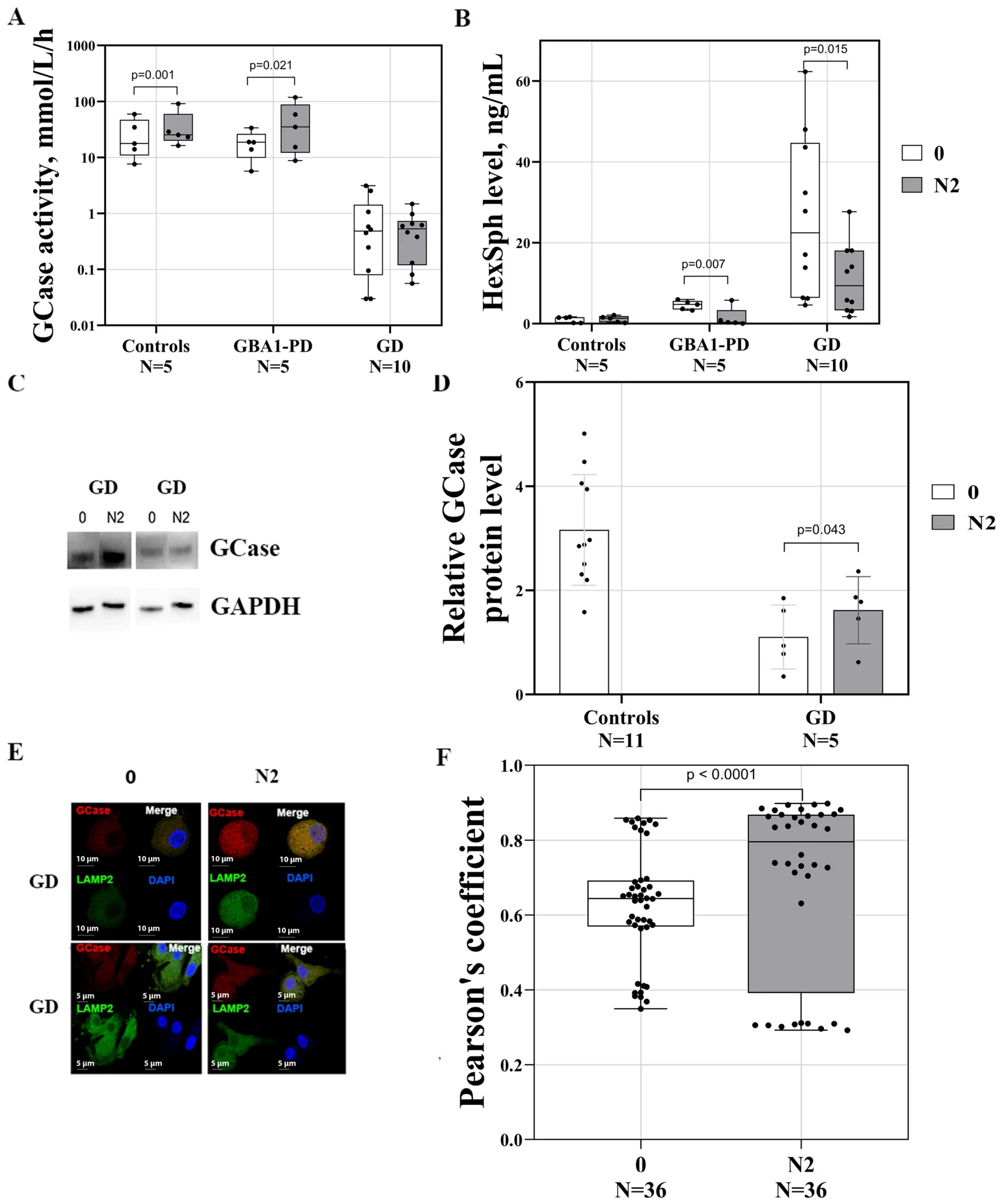
The effects of N2 treatment on PBMC-derived macrophages from GD, GBA1-PD and controls. GCase activity (log scale) (**A**) and HexSph level (**B**) assessed using high-performance liquid chromatography coupled with tandem mass spectrometry (HPLC-MS/MS) in PBMC-derived macrophages (cells concentration 2 × 10^6^ cell/mL) from patients with GD (*n* = 10), GBA1-PD (*n* = 5) (heterozygous *GBA1* mutation (p.N370S or p.L444P) carriers) and controls (*n* = 5) after N2 (4 µM) treatment. Each dot represents an individual data point, with values averaged per individual. GCase protein level (**C**,**D**) in PBMC-derived macrophages from patients with GD (*n* = 5) were visualized on western blot, with GAPDH protein level as the loading control. Each dot represents an individual data point, with values averaged per individual. Colocalization of GCase with the lysosomal marker LAMP2 in PBMC-derived macrophages from patients with GD (*n* = 4) (**E**). Cells were stained for GCase (red) and LAMP2 (green) and visualized using a confocal microscope (×63 magnification), scale bar 5 or 10 µm. Nuclei were counterstained with DAPI (blue). GCase and LAMP2 colocalization (**F**) was quantified using Pearson’s correlation coefficient. Each data point represents the average; “N” indicates the number of fields analyzed (nine fields for each GD patient).

In PBMC-derived macrophages from patients with GBA1-PD, we observed a 1.5-fold increase in GCase activity compared to untreated cells (*p* = 0.021) (Figure 3A). However, in PBMC-derived macrophages from patients with GD, allosteric activator N2 did not affect GCase activity (*p* > 0.05). HexSph level was decreased after treatment with N2 in PBMC-derived macrophages from GD and GBA1-PD patients by 4.14- and 12.1-fold (*p* = 0.007 and *p* = 0.015, correspondingly) (Figure 3B). Interestingly, GCase allosteric activator N2 could affect not only mutant GCase but also the wild-type enzyme. In PBMC-derived macrophages from controls treated with N2, GCase activity was increased by 1.5-fold (*p* = 0.001) (Figure 3A).

At the same time, the effects of N2 treatment on GBA1-PD PBMC-derived macrophages varied depending on the severity of mutations in the *GBA1* gene. Cells from PD patients carrying the p.N370S variant (GBA1-PD3, GBA1-PD4, GBA1-PD5) showed an increase in GCase activity by 2.7-fold after treatment with N2 and a decrease in HexSph level by 25.8-fold (*p* = 0.008 and *p* = 0.010, correspondingly) (Supplementary Figure S1A,B). On the other hand, cells from carriers of the p.L444P variant (GBA1-PD1, GBA1-PD2) when treated with N2, showed a decrease in HexSph level by 4.1-fold (*p* = 0.012), whereas a statistically significant increase in total GCase activity was not detected (Supplementary Figure S1B).

In our study, we investigated the effect of N2 treatment on PBMC-derived macrophages from GD on GCase protein level and its translocation to lysosomes. We showed that treatment with the GCase allosteric activator N2 led to an increase in the relative level of GCase (the ratio of GCase to GAPDH) in GD PBMC-derived macrophages by 1.2-fold (*p* = 0.043) (Figure 3C,D). The degree of colocalization between GCase (red signal) and the lysosomal marker LAMP2 (green signal) was quantified using Pearson’s coefficient in areas of colocalization (Figure 3E,F). N2 treatment significantly increased the translocation of GCase to lysosomes in GD patients (*p* < 0.0001) (Figure 3F).

#### 2.2.2. The Effects of N2 Treatment on iPSC-Derived DA Neurons from GBA1-PD Patients

In this study we used iPSC-derived DA neurons from patients with GBA1-PD and controls to evaluate the effects of the novel GCase allosteric activator N2 on GCase activity and its protein level. The experiments on iPSC-derived DA neurons were carried out in two stages: a primary evaluation in 2022 and a confirmatory replication study in 2025.

iPSC lines previously obtained, characterized and registered in the Human Pluripotent Stem Cell Registry hPSCreg (https://hpscreg.eu) were used for differentiation to DA neurons. Three iPSC lines obtained from one PD patient with the p.N370S mutation in the *GBA1* gene (https://hpscreg.eu/cell-line/ICGi034-A; https://hpscreg.eu/cell-line/ICGi034-B; https://hpscreg.eu/cell-line/ICGi034-C) and one line from each of three apparently healthy individuals (https://hpscreg.eu/cell-line/ICGi021-A; https://hpscreg.eu/cel-lline/ICGi022-A; https://hpscreg.eu/cell-line/ICGi057-A) were taken into the study (all accessed on 20 September 2026).

**Figure 4:**
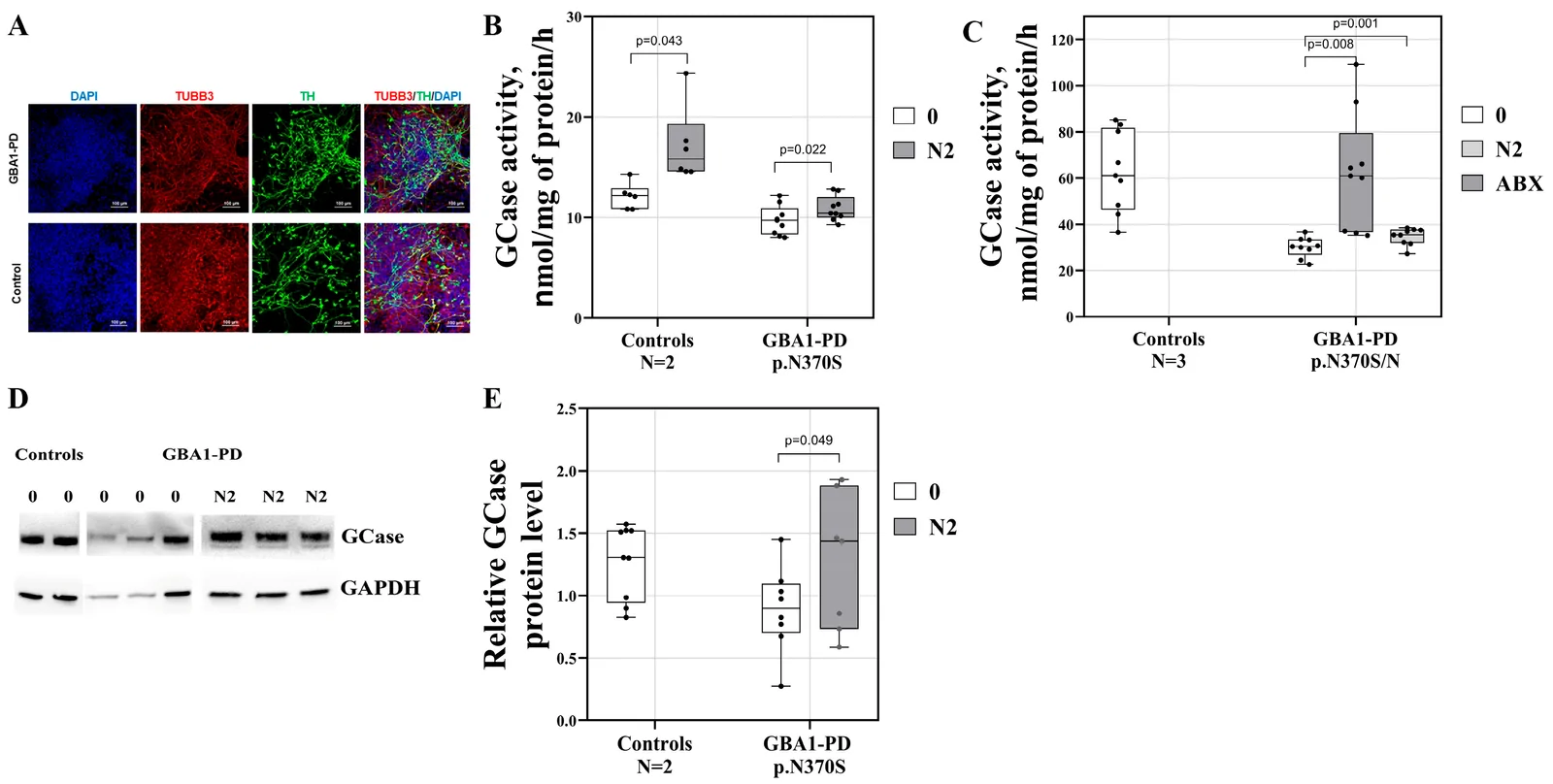
The effects of N2 treatment on iPSC-derived DA neurons from a GBA1-PD patient. Immunofluorescent staining of DA neurons for neuronal markers for TUBB3 (red) and TH (green). Nuclei were counterstained with DAPI (blue). Cells were visualized using a confocal microscope (×63 magnification), scale bar 100 µm (**A**). GCase activity was assessed using HPLC-MS/MS in iPSC-derived DA neurons from a GBA1-PD patient (heterozygous *GBA1* mutation (p.N370S) carrier) after treatment with N2 (4 µM, 21 days), assessed in a primary evaluation in 2022 (**B**) and in a confirmatory replication study in 2025 (**C**). (**B**) represents data from iPSC-derived dopaminergic neurons obtained from the GBA1-PD: three independent iPSC lines from one individual, each with three biological replicates analyzed in three technical replicates. The controls include two independent iPSC lines from two individuals, each with three biological replicates analyzed in three technical replicates. (**C**) represents data from iPSC-derived dopaminergic neurons obtained from the GBA1-PD: three independent iPSC lines (from one individual), each with three biological replicates analyzed in three technical replicates. The controls include three independent iPSC lines (from three individuals), each with three biological replicates analyzed in three technical replicates. GCase protein level in iPSC-derived DA neurons from a GBA1-PD patient (**D**,**E**) were visualized on western blot, with GAPDH protein level as the loading control. Each dot represents an individual data point.

The differentiation efficiency was assessed by immunofluorescent staining for markers of DA neurons—tyrosine hydroxylase (TH) and neural marker β3-tubulin (TUBB3) (Figure 4A).

In this study, we demonstrated that treatment with the novel GCase allosteric activator N2 resulted in a 1.15-fold increase in GCase activity (*p* = 0.022) and a 1.4-fold increase in GCase relative protein level (*p* = 0.049) in DA neurons derived from GBA1-PD patients (Figure 4B,D,E). Additionally, we observed a 1.2-fold increase in GCase activity in iPSC-derived DA neurons from controls after N2 treatment (*p* = 0.043) (Figure 4B).

In addition, we conducted a replication study to evaluate the efficacy of N2 in a separate experiment. Furthermore, we compared the efficacy of the allosteric GCase activator N2 with that of the well-established pharmacological chaperone ABX on DA neurons derived from a patient with GBA1-PD. We have identified that both ABX and N2 increased GCase activity in a patient with GBA1-PD by 2.0- and 1.16-fold, correspondingly (*p* = 0.008 and *p* = 0.011, correspondingly) (Figure 4C).

Thus, we confirmed the effect of the novel GCase allosteric activator N2 on GCase enzymatic activity in iPSC-derived DA neurons from a GBA1-PD patient.

#### 2.2.3. The Effects of N2 Administration on GCase Activity, HexSph Level, DA Level and Neurodegeneration in Mice

In this part of the study, we assessed GCase activity and HexSph level in the cortex and midbrain of wild-type C57BL/6 mice, as well as in the cortex of mice with MPTP-induced PD that received a novel allosteric GCase activator N2 and ABX via intraperitoneal injection. The MPTP-induced mouse model of early symptomatic PD was generated according to the previously reported protocol [56]. To confirm the development of neurodegeneration in this model, we evaluated dopamine level in the striatum and quantified the number of TH^+^ neurons in substantia nigra (SN).

##### The Effects of N2 on GCase Activity and HexSph Level in Wild-Type C57BL/6 Mice

**Figure 5:**
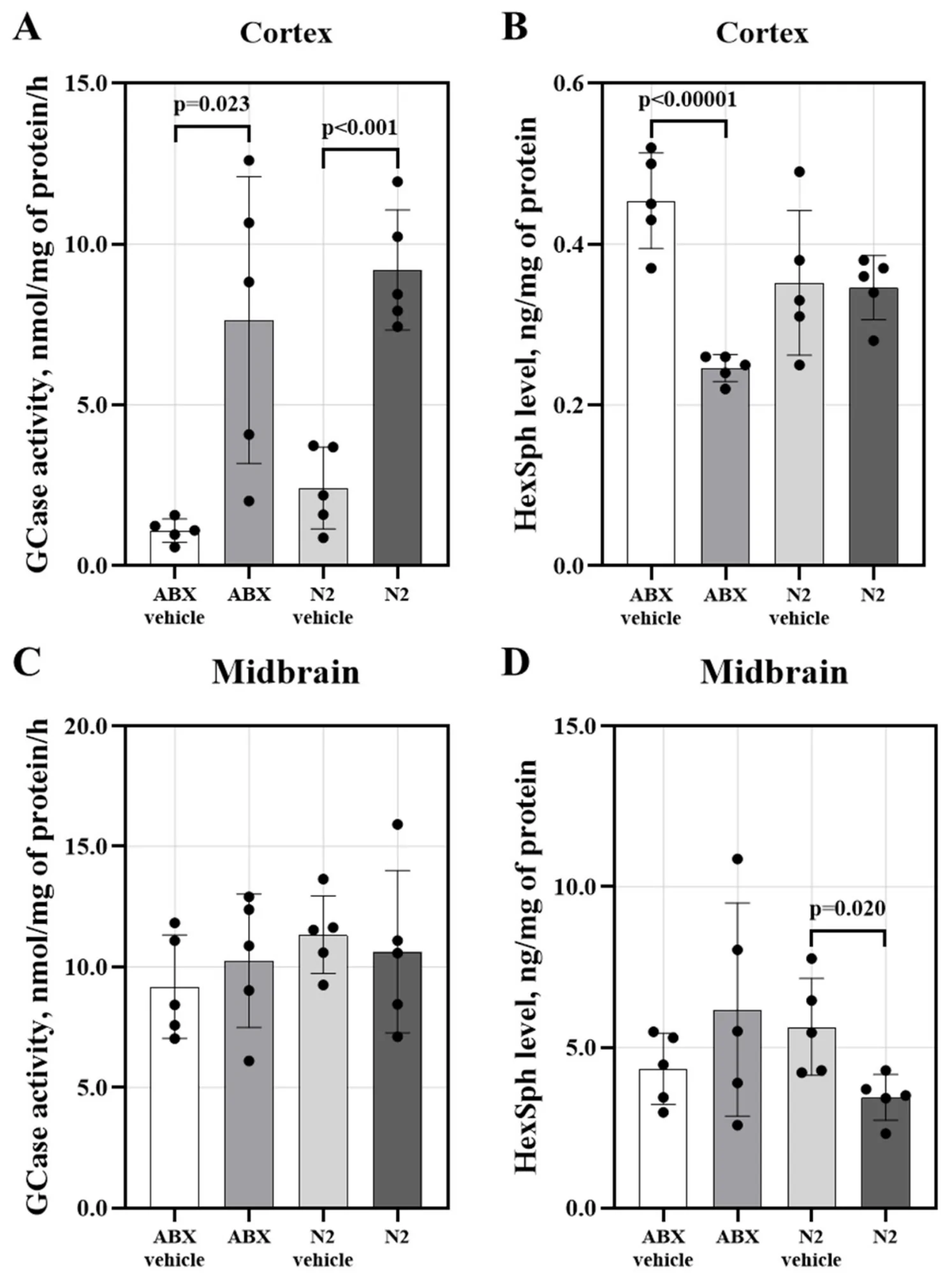
The effects of GCase activators (N2, ABX) on GCase activity (**A**,**C**) and HexSph level (**B**,**D**) assessed using HPLC-MS/MS in brain regions (prefrontal cortex, midbrain) of wild-type C57BL/6 mice (*n* = 5 mice per group).

Administration of N2 produced a 4-fold increase in GCase activity in the prefrontal cortex compared to vehicle-treated mice (*p* < 0.001) (Figure 5A). ABX administration led to a significant 8-fold increase in enzyme activity in the same brain region compared to vehicle-treated mice (*p* = 0.023) (Figure 5A). In contrast, neither compound affected GCase activity in the midbrain (*p* > 0.05) (Figure 5C). Compared to vehicle-treated mice, N2 treatment significantly reduced HexSph level specifically in the midbrain (*p* = 0.02) (Figure 5D). ABX administration resulted in a marked decrease in HexSph level in the prefrontal cortex compared to the vehicle-treated group (*p* < 0.0001) (Figure 5B).

##### The Effects of N2 on GCase Activity, HexSph Level, Dopamine Level and Neurodegeneration in an MPTP-Induced Mouse Model of PD

**Figure 6:**
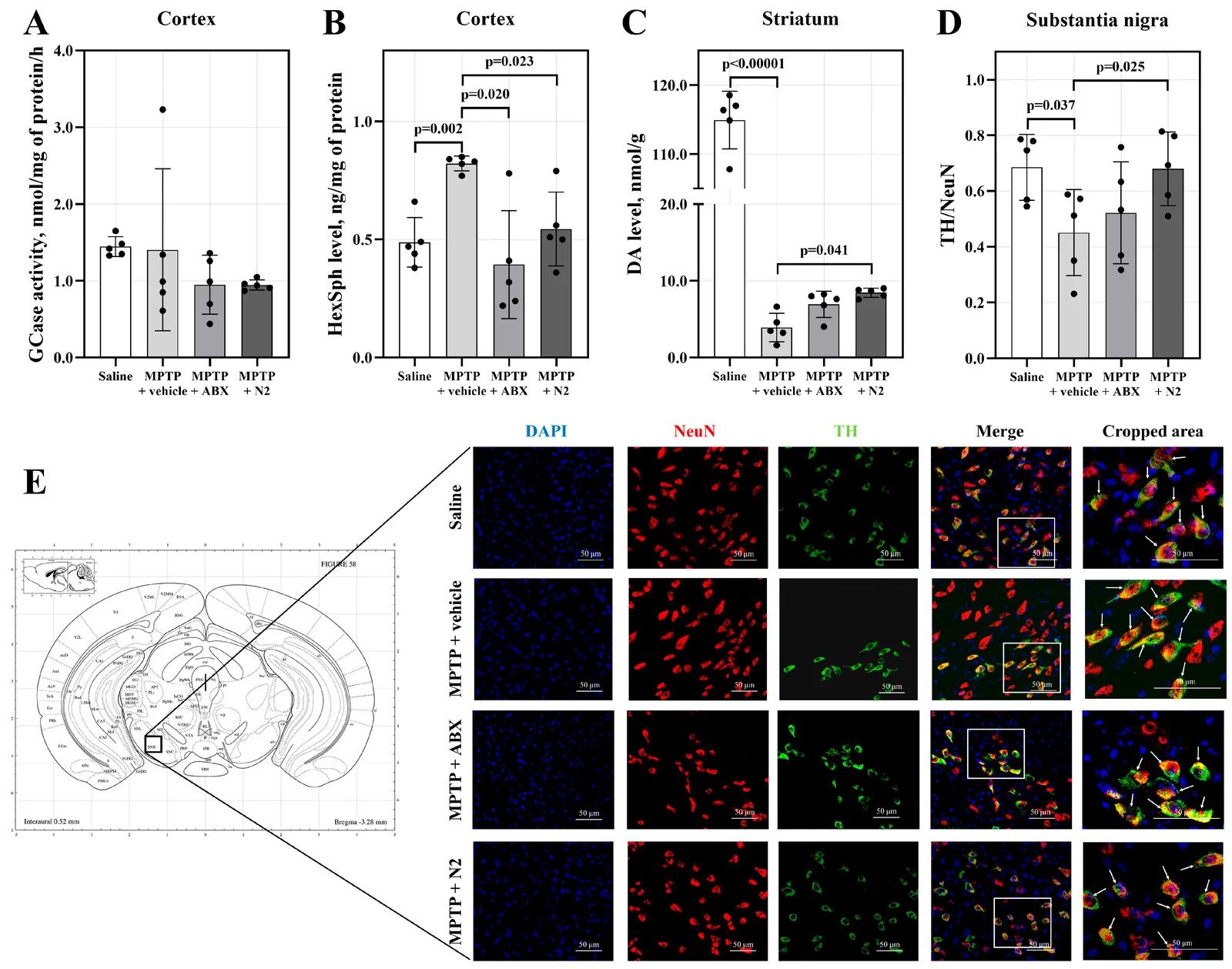
Assessment of GCase activity, sphingolipid homeostasis, and dopaminergic integrity following N2 and ABX administration in MPTP-treated mice. Evaluation of GCase activity (**A**) and HexSph level (**B**) in the prefrontal cortex using HPLC-MS/MS, alongside striatal dopamine level (**C**) using high-performance liquid chromatography (HPLC), and dopaminergic neuron loss in the SN (**D**,**E**) (*n* = 5 mice per group). The density of surviving DA neurons was quantified by counting TH and NeuN colocalized cells via immunofluorescence double-staining. (**D**) The percentage of surviving TH^+^ neurons in the SN (*n* = 5 mice per group). (**E**) Representative images of the SN cryosections stained for TH (green), NeuN (red), and DAPI (blue), obtained using confocal microscopy (magnification ×63; scale bar, 50 µm). Insets show higher-magnification views of the selected areas, indicated by white boxes. White arrows indicate TH^+^ neurons. The anatomical schematic on the left was adapted using the online Mouse Brain Atlas tool (gaidi.ca) [57].

As expected, MPTP administration (4 × 12 mg/kg) in mice led to a significant 28.5-fold decrease in striatal dopamine level (*p* < 0.00001) and a 1.4-fold reduction in the number of TH^+^ neurons in the SN (*p* = 0.037) compared to control mice (Figure 6C,D). Interestingly, administration of MPTP (4 × 12 mg/kg) also led to an increase in HexSph level in the prefrontal cortex compared to control mice (*p* = 0.002), whereas GCase activity remained unchanged (*p* > 0.05) (Figure 6A,B).

In the same MPTP-induced PD mice, administration of the GCase activators N2 and ABX led to a significant decrease in HexSph level in the prefrontal cortex compared to vehicle-treated mice (N2, *p* = 0.023, ABX, *p* = 0.02) (Figure 6B). Interestingly, GCase activity did not differ in the prefrontal cortex across all study groups (*p* > 0.05) (Figure 6A). Concurrently, in the prefrontal cortex of wild-type mice, N2 altered GCase activity but had no effect on HexSph level. Crucially, mice with MPTP-induced PD were sacrificed 24 h after the last injection, whereas wild-type mice were sacrificed one hour after the injection. Thus, these data may indicate that enzyme activity is a more dynamic parameter than substrate level.

Only N2, an allosteric GCase activator, when administered after MPTP, produced an increase in striatal dopamine level compared to the MPTP-vehicle group (*p* = 0.041) (Figure 6C). ABX treatment also increased dopamine level, but the difference was not statistically significant (*p* > 0.05) (Figure 6C). The number of TH^+^ neurons in the SN of mice that administered the allosteric GCase activator N2 was increased compared to the MPTP+vehicle group (*p* = 0.025) (Figure 6D,E).

## 3. Discussion

In the present study, we introduced a novel allosteric GCase activator, N2, and demonstrated its efficacy in both patient-derived cells (PBMC-derived macrophages and iPSC-derived DA neurons) from GD and GBA1-PD patients and in a mouse model of MPTP-induced parkinsonism, where N2 successfully prevented neurodegeneration. To the best of our knowledge, this is one of the first studies to validate the therapeutic potential of a newly synthesized allosteric GCase activator across both complex human cellular models and in vivo neurotoxic models. Our findings underscore the critical role of GCase activation as a viable strategy to counteract lysosomal dysfunction and subsequent neuronal loss. These results not only predict binding mode of N2 with its target but also provide compelling evidence for its systemic neuroprotective effects, which are discussed below.

Although the association between *GBA1* mutations (even in the heterozygous state) with an increased risk of PD development is well established, *GBA1* mutations can be classified according to their impact on the severity of GD and residual GCase enzymatic activity: “mild” variants (e.g., p.N370S) and “severe” variants (e.g., p.L444P, p.c84dupG) [9]. Different *GBA1* mutations associated with GD phenotype may have different effects on PD risk [9]. The odds ratios for developing PD with “mild” p.N370S and “severe” p.L444P mutations range from 1.8 to 7.8 and 2.6 to 30.4, respectively, making it difficult to predict which patients will develop PD [12,58]. Therefore, as part of our study, we examined how N2 affects GCase activity and HexSph level depending on the type of mutation in the *GBA1* gene in patients with PD. However, the molecular basis of this association has not yet been fully elucidated. Different mechanisms have been proposed. First, *GBA1* variants linked to PD are associated with decreased GCase activity [11,17,20,26,30,59], suggesting a loss-of-function effect. Next, severe variants (p.L444P) and to a lesser extent the mild p.N370S variant, cause GCase misfolding in the ER, leading to its substantial retention there and subsequent ER stress [11,22,29,52,60,61,62]. Both mechanisms could contribute to the pathogenesis of the disease. However, in this regard therapeutic approaches such as gene therapy, which is currently being tested in the ongoing clinical trials (NCT04127578), might be less effective than the PC strategy, which promotes the correct folding and trafficking of GCase to the lysosomes.

To date, the most promising agent is ABX, a mixed-type GCase PC (with inhibitory activity at neutral pH) [40,43]. ABX is blood–brain barrier-permeable and well tolerated [63], and has shown favorable results in pilot studies for neuronopathic GD. Clinical trials are currently underway for its application in PD and dementia with Lewy bodies (NCT06193421, NCT04388969, NCT05830396, NCT05778617, NL-OMON52316, CTIS2024-519088-16-01, ACTRN12623000843651, NL-OMON51936, EUCTR2021-006224-40-PL, KCT0003218, EUCTR2021-002550-82-NL, JPRN-jRCTs061190017, JPRN-UMIN000009392, JPRN-JMA-IIA00421, NCT05778617, NCT04405596, NCT05830396, NCT06193421, NCT05773443, NCT02914366, NCT0458825) [64,65,66,67,68,69]. Although ABX has demonstrated efficacy in cell-based and animal models, as well as in clinical trials, allosteric GCase activators are believed to offer distinct advantages due to their selective action on GCase.

Previously, using molecular docking combined with molecular dynamics simulations, we identified and characterized six allosteric pockets on the GCase surface suitable for the binding of enzyme activators; among them, the BS1 site proved to be energetically preferred for the NCGC607 and is located in close proximity to the catalytic center of the enzyme [55]. The BS1 pocket is delimited by Loops 6 and 7 and α-helix 7 and is partially formed by the cavity that arises as a direct structural consequence of the p.N370S amino acid substitution, which makes this site specific to the mutant enzyme. Since BS1 lies immediately underneath Loop 6, whose structural stability is known to affect the catalytic activity of GCase [70], the accommodation of a ligand within this pocket may stabilize Loop 6, compensate for the local packing defect introduced by the p.N370S substitution and affect the mobility of the adjacent catalytic nucleophile Glu340. Because the compounds are bound outside the substrate-binding cleft, this mode of action is conceptually distinct from that of active-site-directed iminosugar chaperones and is not expected to be accompanied by competitive inhibition of the enzyme.

Here, molecular docking and MD simulations predict that N2, a derivative of the previously described non-inhibitory GCase chaperone NCGC607, can be accommodated in the BS1 allosteric pocket of p.N370S GCase (Figure 2). These in silico findings provide a structural hypothesis for a BS1-directed mechanism. Compounds N2 and NCGC607 share a common 2-[2-(4-iodoanilino)-2-oxoethoxy]benzoyl scaffold and are accommodated in BS1 in closely related orientations: the three structural blocks constituting both molecules occupy essentially equivalent spatial positions, with the 4-iodophenyl fragment buried in the hydrophobic subcavity enclosed by Loops 6 and 7 and α-helix 7, the central benzene ring with its oxyacetamide linker aligned along the pocket axis, and the terminal aromatic moiety directed towards the region adjacent to residue 370 and Loop 6. The two compounds differ, however, in the way their complexes are stabilized. In the BS1.1 pose, NCGC607 is anchored by hydrogen bonds involving Asp315, Ser366, Gln362 and Lys346. In contrast, N2 is retained by a hydrogen network formed with Arg285, Asp315, and Ser370. This rearrangement of the hydrogen-bonding pattern is reflected in the binding energetics: the MM/GBSA binding free energy of N2 (−46.69 ± 7.95 kcal/mol) is somewhat smaller in absolute magnitude than the value obtained earlier for the BS1.1 pose of NCGC607 (−53.7 ± 4.0 kcal/mol) [55]. Given that MM/GBSA estimates lacking an explicit solute configurational entropy term are suitable for the relative ranking of ligands rather than for the prediction of absolute affinities, both compounds can be regarded as belonging to the same affinity range.

Here, we present evidence that N2, a derivative of the previously described allosteric GCase activator NCGC607, is potentially binds to GCase (Figure 2). N2 enhances GCase lysosomal translocation (Figure 3), modulates its activity and protein level, and decreases substrate accumulation in patient-derived cells. Our results demonstrate that N2 acts as an effective GCase activator. The small-molecule GCase enhancer NCGC607 was initially identified through a high-throughput screening of chemical libraries conducted on spleen tissue homogenates from a GD patient (p.N370S/p.N370S) by Ellen Sidransky and colleagues [71,72]. NCGC607 successfully restored GCase activity and protein level, while reducing substrate level in both iPSC-derived macrophages and DA neurons from GD patients, including neuronopathic forms. Additionally, it reduced alpha-synuclein level in DA neurons from GBA1-PD patients, suggesting that NCGC607 holds therapeutic potential for both GBA1-PD and neuronopathic GD [72,73].

Recently, the same researchers developed a high-throughput cell-based luminescence assay utilizing GCase labeled with a small proluminescent peptide, which allows for the measurement of protein stabilization, and expressed this fusion protein in neuroglioma cells [52]. The conducted screening revealed more effective analogs of NCGC607, designated as NCGC326 [52]. The efficacy of NCGC326 was validated in fibroblasts from healthy controls and GD patients (p.N370S/p.N370S).

In our recent studies, we also demonstrated the effectiveness of both ABX and NCGC607 in restoring GCase activity in patient-derived cells [43,44,55]. Comparing the reported efficacy of ABX and NCGC607 [43,44,55] with the results obtained for N2 in the present study, compound N2 demonstrated a capacity to restore GCase activity in patient-derived cells from GD and GBA1-PD patients that was broadly comparable to these agents.

Previously, using patient-derived macrophages, we demonstrated that NCGC607 increased GCase activity in PBMC-derived macrophages from GD patients by 1.3-fold, but not in those with GBA1-PD [55]. In contrast, N2 increased GCase activity exclusively in PBMC-derived macrophages from GBA1-PD patients by 1.5-fold. Furthermore, NCGC607 promoted a significant reduction in HexSph level in PBMC-derived macrophages from GD patients by 6.84-fold, but had no impact on the GBA1-PD group. However, N2 reduced HexSph level both in GD patients by 4.17-fold and in GBA1-PD patients by 12-fold. In addition, we showed that N2 increased the lysosomal transport of GCase in PBMC-derived macrophages from GD patients. In the control group, NCGC607 and N2 increased GCase activity by 2.1-fold and 1.5-fold, respectively. A comparative analysis revealed that compound N2 exhibited a superior therapeutic and pharmacological profile compared to NCGC607, especially in the context of GBA1-PD. Taken together, our preliminary data suggest that NCGC607 analogs may represent a potential class of allosteric-site-directed GCase activators.

It should be noted that beyond ABX, to the best of our knowledge, three small-molecule allosteric GCase activators are currently being evaluated in ongoing clinical trials: BIA 28-6156 (LTI-291) (NCT05819359), GT-02287 (NCT06732180) and VQ-101 (EUCT number: 2023-509158-68-00, 2026-525848-15-00) [29,53,54]. BIA 28-6156 (LTI-291) [53] is the derivative of the compound NCGC758, whose potency to increase GCase activity was initially detected via chemical screening conducted on the spleen tissue homogenates from a GD patient (p.N370S/p.N370S) [74,75]. GT-02287 was identified by means of the SEE-Tx* computational drug discovery platform in the frame of an international consortium [76]. GT-02287 has been shown to enhance GCase enzyme activity and protein level, reduce substrate accumulation, and restore the lysosomal transport of GCase in fibroblasts from both healthy controls and GD patients, including neuronopathic forms. In addition, GT-02287 attenuated ER stress in patient fibroblasts [76].

However, no data have yet been published on the neuroprotective properties of allosteric GCase modulators in animal models.

To date, the exact mechanisms driving neurodegeneration in PD remain poorly understood. As a result, current therapeutic strategies offer only symptomatic relief, highlighting an urgent need for disease-modifying approaches capable of delaying disease progression. Our results, alongside findings from other studies, demonstrate that GCase activity is reduced in the blood and brain tissue of iPD patients, suggesting that wild-type GCase could also serve as a potential therapeutic target. Here, we present the first assessment of the effects of the allosteric GCase activator N2 on GCase activity in the brains of wild-type mice and a mouse model of MPTP-induced parkinsonism, comparing its action with that of ABX.

To date, various models of induced parkinsonism have been described [77,78]. The MPTP- and 6-hydroxydopamine (6-OHDA) -induced parkinsonism mouse models are the gold standard for neurotoxic animal models of PD and are widely used to evaluate neuroprotective activity in drug development [79,80,81]. The neuroprotective effect of ABX was first demonstrated in rats with 6-OHDA-induced loss of nigral cells [48]. Notably in the study by Mishra [48], rats with 6-OHDA-induced loss of DA neurons exhibited a decrease in GCase activity first, suggesting that neurotoxic iPD models could reproduce the decrease in GCase activity described in the brains of iPD patients who do not carry *GBA1* mutations [30,31,32,33,34,82]. Consistently, a decrease in GCase activity along with an increase in GluSph level was detected in specific brain regions of rats after five days of rotenone dosing [83]. This is in accordance with our present data, as we reported here for the first time that MPTP injection (4 × 12 mg/kg) leads to an increased level of HexSph in the prefrontal cortex of the mouse brain. Interestingly, we failed to detect any alterations in GCase activity in the mouse brain after a double MPTP injection (2 × 12 mg/kg) [84], indicating that MPTP treatment could exuberate GCase activity in the brains of model animals in a dose-dependent manner. Moreover, these data may also indicate that enzyme activity is a more dynamic parameter than the substrate level. Interestingly, the blood HexSph level is considered a more sensitive marker than GCase activity [20].

Overall, the data obtained from different neurotoxic animal models suggest that GCase disturbance are not limited to genetic models of parkinsonism carrying pathogenic *GBA1* variants but are also relevant to iPD models. This indicates that neurotoxic animal models of parkinsonism could be used to evaluate the efficiency of compounds targeting GCase activity.

Here we demonstrated for the first time that the allosteric GCase activator N2 leads to a decrease in HexSph levels, the amelioration of TH^+^ neuronal loss in the SN and a reduction of dopamine levels in the striatum in MPTP neurotoxic models of parkinsonism in mice.

The capacity to modulate GCase activity in the brains of wild-type mice was previously demonstrated for two allosteric GCase activators: S-181, an analog of the compound NCGC758 [51], and another novel allosteric GCase activator (compound 3), which was identified through an innovative computational approach for in silico screening of small-molecule allosteric modulators [85]. Similar to our findings with compound N2, S-181 treatment in mice resulted in an increase in GCase activity in the brain tissue and led to a reduction in GCase lipid substrates [51]. For compound 3, only its brain penetration was assessed [85]. To date, no study has compared the action of allosteric GCase modulators with that of ABX in animal models, and the ability of allosteric GCase modulators to protect DA neurons from degradation remains to be reported.

**Our study has potential limitations.** First, this was an exploratory study with a relatively small sample size. Second, further experimental investigations are required to directly evaluate the blood–brain barrier permeability of N2 in vivo. Third, molecular docking, MD simulations, and MM/GBSA calculations identify a computationally plausible N2 pose in the BS1 pocket of p.N370S GCase, but do not demonstrate direct physical binding and binding affinity. These issues should be addressed using purified GCase in biophysical assays, such as surface plasmon resonance, microscale thermophoresis, isothermal titration calorimetry and differential scanning fluorimetry. Fourth, because the current structural analysis was restricted to p.N370S GCase, it does not explain the molecular basis of the functional response observed in p.L444P carrier-derived macrophages. Furthermore, the therapeutic efficacy of N2 should be comprehensively benchmarked not only against established GCase PCs, such as ABX, but also against novel GCase allosteric activators, particularly those currently undergoing evaluation in ongoing clinical trials. The question remains if allosteric GCase activators could also bind to the non-lysosomal glucosylceramidase encoded by *GBA2*. Measuring GCase activity in the whole cell lysates (total) instead of purified lysosomal fractions significantly limits accuracy by including inactive enzyme precursors, failing to reflect pathogenic trafficking defects, and missing changes in the acidic lysosomal microenvironment, often masking functional deficiencies. In addition, the lack of a direct dose-dependent relationship between residual GCase activity and PD risk—as evidenced by low PD penetrance in GD patients versus heterozygous carriers—shifts focus toward a gain-of-function paradigm, such as mutant GCase retention and subsequent ER stress [29]. Since our study did not directly measure the unfolded protein response cascade, future research is needed to investigate whether N2 can alleviate this proteotoxic ER stress or if its therapeutic efficacy is restricted to the lysosomal compartment.

A limitation of this study is also that behavioral tests, such as the rotarod, open-field, or elevated plus-maze tests, were not performed on the MPTP-induced mouse model. Consequently, further in vivo behavioral studies are required to fully confirm the neuroprotective functional outcomes of the evaluated N2 GCase activator.

## 4. Materials and Methods

### 4.1. Molecular Modeling

Molecular modeling was performed using computational resources of Peter the Great Saint-Petersburg Polytechnic University Supercomputing Center.

#### 4.1.1. Construction of N2 Atomistic Model

The atomistic model of the p.N370S mutant form of GCase was prepared for docking using a protocol that we described in detail previously [43]. A crude spatial model of the N2 molecule (2-(2-(4-iodophenylamino)-2-oxoethoxy)benzoyl-N-phenylhydrazine carboxamide) was built based on the NCGC00241607 compound obtained from the PubChem database (PubChem CID 46943210; accessed on 10 September 2026) using the Molsoft ICM-Pro molecular editor (version 3.8; MolSoft, San Diego, CA, USA) [86]. The geometry of the constructed compound was optimized in the GAMESS (US) program (version 2024 R2; Iowa State University, Ames, IA, USA) [87] using the DFT B3LYP method and the 3-21G basis set. Water solvent effects were included using the polarizable continuum model (PCM). The multiplicity of the electronic state of the molecules was regarded as 1, and the charge state was neutral.

#### 4.1.2. Molecular Docking

Molecular docking simulations were performed using the Molsoft ICM-Pro software package [86]. The docking of compound N2 was carried out into the allosteric binding site BS1, which we identified previously [43,55]. To take into consideration the flexibility of the binding sites, we generated conformational ensembles of the protein via MD simulations. The obtained GCase structures were clustered by the positions of the amino acid residues forming the BS1 binding site. The median structures of each cluster were chosen for an ensemble docking procedure known as four-dimensional docking (4D docking) [88]. Four-dimensional docking accounts for the conformational variability of the receptor in a single docking simulation and reduces the sampling time while preserving the accuracy of traditional ensemble docking. Docking results were filtered according to the values of the scoring function, and five distinct poses of the N2 compound were manually selected for refinement and stability testing via 50 ns MD simulations.

#### 4.1.3. Molecular Dynamics Simulations

Simulations in the explicit solvent were carried out using the AMBER software suite (version 22; University of California, San Francisco, CA, USA) [89], using the AMBER ff14SB force field to account for the protein [90] and the GAFF2 force field for N2. Structures of the enzyme or its complexes with N2 were placed in an octahedral box of TIP3P water, with the box edge at least 12 Å away from the solute at all points. Fifteen Cl− ions were added to neutralize the system. MD simulations were carried out according to a standard protocol, including: (1) a two-stage minimization, where the first stage predominantly affected the coordinates of the solvent molecules while the positions of protein/N2 atoms were restrained (force constant of 1.0 kcal/(mol⋅Å^2^), and the second stage affected the entire system; (2) gradual heating of the system from 0 K to 310 K over 100 ps, keeping the protein/N2 atoms restrained (force constant of 1.0 kcal/(mol⋅Å^2^)); (3) 1 ns equilibration of the system with attenuated restraints on the protein/N2 atoms (force constant of 0.1 kcal/(mol⋅Å^2^), followed by 1 ns equilibration of the unrestrained system; and (4) a 50 ns productive run.

A Langevin thermostat with a collision frequency of γ = 2 ps^−1^ was used for temperature control at 310 K. Constant pressure was maintained using an isotropic scaling and a Berendsen barostat with a pressure relaxation time of 2 ps. Long-range electrostatics were treated using the particle mesh Ewald (PME) method [91]; non-bonded Coulomb and Lennard-Jones interactions were truncated at 9 Å. The molecular dynamics integration step was 2 fs. All covalent bonds involving hydrogen atoms were constrained using the SHAKE algorithm. Trajectories were processed using the CPPTRAJ module of AMBER. The last 25 ns of the molecular dynamics trajectories were used for the analysis. Cluster analysis was used to identify the most populated conformations of the enzyme polypeptide chain and N2 in the binding pockets. The MM/GBSA method (Generalized Born model igb = 5 and a salt concentration of 0.15 M) was used to evaluate the binding free energies of N2.

### 4.2. The Chemical Synthesis of N2 and Its Characterization

**Figure 7:**
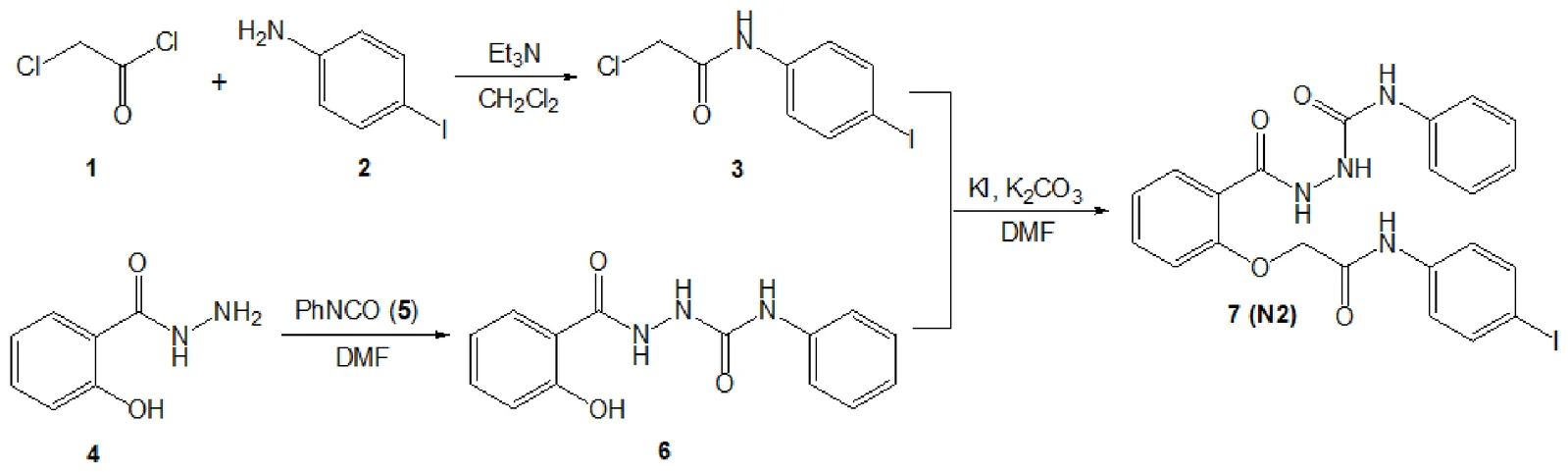
N2 synthesis scheme. 1: chloroacetyl chloride, 2: 4-iodoaniline, 3: 2-chloro-N-(4-iodophenyl)acetamide, 4: salicyloylhydrazide, 5: phenyl isocyanate, 6: 2-(2-hydroxybenzoyl)-*N*-phenylhydrazinecarboxamide, 7 (N2): 2-(2-(2-((4-Iodophenyl)amino)-2-oxoethoxy)benzoyl)-*N*-phenylhydrazinecarboxamide (7 (N2)).

The synthesis scheme of N2 is illustrated in Figure 7. Detailed chemical synthesis of N2 and its characterization (1H-NMR spectra, 13C-NMR spectra, and HRMS data) are described in detail in the Supplementary Materials.

### 4.3. Patients

The study included patients with GD (*n* = 10, mean age 39.5 ± 3.7 years, 50% men), GBA1-PD (PD patients with heterozygous mutations in the *GBA1* gene) (*n* = 6, mean age 55.5 ± 3.5 years, 17% men), and healthy volunteers (*n* = 7, mean age 41.3 ± 4.2 years, 42% men) (Table 2).

The study was conducted in accordance with the guidelines of the Declaration of Helsinki. Biomaterial was collected at Pavlov First Saint-Petersburg State Medical University with the permission of the ethics committee (Protocol number: 244, Approval Date: 25 January 2021), N.P. Bechtereva Institute of the Human Brain RAS (Protocol No. 1, 26 November 2020). Written informed consent or assent was obtained from patients prior to sample collection.

PD diagnosis was confirmed based on published criteria [92]. The diagnosis of GD was confirmed by measuring GCase activity in leukocytes, while the patient’s genotype was established by sequencing of all exons of the *GBA1* gene. Blood samples from GD patients were taken at least 2 weeks after the last enzyme replacement therapy infusion.

GD and PD patients were enrolled at the Almazov National Medical Research Center, Pavlov First Saint-Petersburg State Medical University, Institute of the Human Brain of RAS. The control group was recruited at the Pavlov First Saint-Petersburg State Medical University. *GBA1* variants (p.N370S and p.L444P) were confirmed in PD patients and excluded in controls by Sanger sequencing.

### 4.4. PBMC-Derived Macrophages and N2 Treatment

Differentiation of PBMCs to macrophages from freshly isolated PBMCs (obtained by Ficoll density gradient centrifugation (Ficoll-Paque PLUS, GE Healthcare, Chicago, IL, USA) was carried out in RPMI1640 medium (Corning, NY, USA) supplemented with 10% FBS (Corning, Glendale, AZ, USA) using macrophage colony-stimulating factor (M-CSF) (10 ng/mL) (BioLegend, San Diego, CA, USA) and incubated at 37 °C in 5% CO_2_ for 4 days [93]. To determine the optimal N2 concentration with minimal cytotoxicity, a colorimetric MTS assay (CellTiter 96 AQueous One Solution; Promega, Madison, WI, USA) was performed (Supplementary Figure S2). To investigate the effects of novel allosteric activator N2, PBMC-derived macrophages were cultured in a complete medium containing 4 μM of N2 for 4 days.

### 4.5. iPSC-Derived DA Neurons and N2 Treatment

Generation of iPSCs from patients’ PBMCs, culturing and their detailed characterization were carried out according to the previously described protocol [44,94].

Directed DA neuron differentiation of iPSCs was carried out on Matrigel-GFR (Corning, Glendale, AZ, USA)-coated dishes according to previously published procedures with some modifications [44,95]. On day 11 of differentiation, the cell monolayer was passaged using Accutase (Gibco, Thermo Fisher Scientific, Waltham, MA, USA). On the next day, BDNF, GDNF, TGFb3 and dbcAMP (all PeproTech, Cranbury, NJ, USA) were added to the growth medium. The cells were passaged weekly. After 30 days, the cells were transferred to new wells at a density of 1–2 × 10^5^ cells/cm2 in the medium containing 0.1 µM Compound E (Sigma-Aldrich, St. Louis, MO, USA). Immunofluorescent staining of cells for specific markers of DA neurons (antibodies are listed in the Supplementary Table S1) was performed according to the previously described procedure [96]. Sixty-day-old iPSC-derived DA neurons were used for experiments. To investigate the effects of novel allosteric activator N2, DA neurons derived from patient iPSCs were cultured in a complete medium containing 4 μM of N2 for 21 days.

### 4.6. Animals

Male C57BL/6 mice aged 7–10 weeks (8–10 week-old for the first stage of the study, 7–8 week-old for the second stage of the study) and weighing 22–27 g were used. Animals were housed under standard conditions (21–23 °C, 12 h light/dark cycle) with free access to food and water in the vivariums of NRC “Kurchatov Institute”—PNPI or NRC “Kurchatov Institute”. Mice were obtained from specific pathogen-free facilities of the Institute of Cytology and Genetics, Siberian Branch of the Russian Academy of Sciences (stage 1 of the animal studies) and the Experimental Biological Clinic of the Kurchatov Complex of NBICS-Nature-like Technologies, National Research Center “Kurchatov Institute” (stage 2 of the animal studies). All animal procedures were performed in accordance with the Guide for the Care and Use of Laboratory Animals [97] and were approved by the Ethics Committees of PNPI (Protocol No. 5.9.24, 3 September 2024) and the Kurchatov Institute (Protocol No. 1, 16 January 2024).

### 4.7. Experimental Design and Pharmacological Treatment

**Figure 8:**
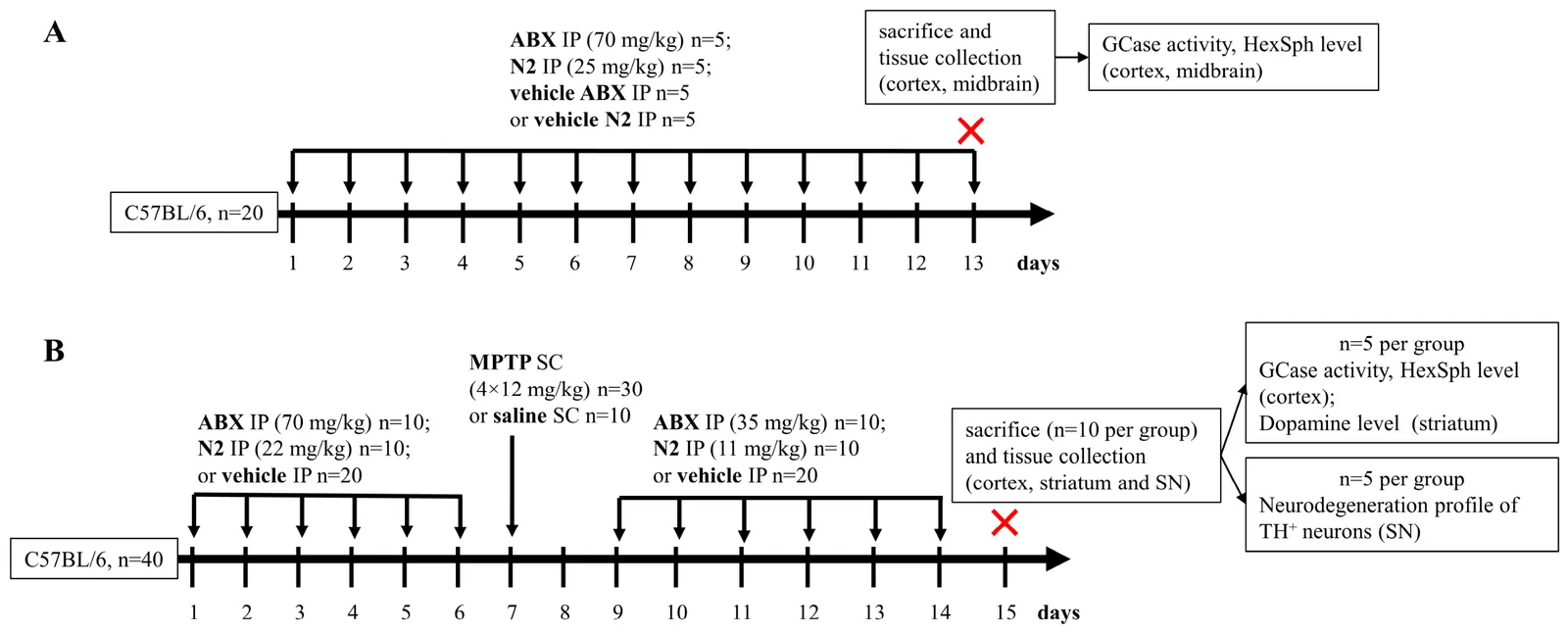
Experimental design for evaluating the effects of allosteric GCase activators (N2 and ABX) in wild-type C57BL/6 mice (**A**) and an MPTP-induced mice model of PD (**B**). Intraperitoneal injections (IP), subcutaneous injections (SC), 1-methyl-4-phenyl-1,2,3,6-tetrahydropyridine (MPTP), tyrosine hydroxylase positive (TH^+^), substantia nigra (SN).

The experimental project consisted of two stages and is shown in Figure 8.

No formal a priori sample size calculation was performed. All animals that completed the experimental protocol were included in the analysis. Animals showing signs of illness or improper injection would have been excluded; however, no animals met any of these exclusion criteria in the present study. Animals were randomly allocated into control and treatment groups based on their body weight, using a randomization sequence generated by the RAND function in Microsoft Excel (version 2021; Microsoft Corp., Redmond, WA, USA). Potential confounders, such as the order of treatments and measurements, or animal/cage location, were not explicitly controlled. However, all groups were handled identically and under the same environmental conditions. The investigator conducting the experiment and administering treatments was blinded to group allocation. Outcome assessment and data analysis were performed by a blinded investigator. To ensure animal welfare, general health was assessed daily by monitoring appearance, posture, mobility, grooming, body condition, and signs of pain or distress. Specific humane endpoints were predefined in accordance with the approved animal experimentation protocol, with immediate euthanasia planned for any animal meeting these criteria. However, no animals reached any humane endpoint during the study.

#### 4.7.1. The Wild-Type C57BL/6 Mice

For stage 1 of the animal studies, the mice were divided into 4 groups (*n* = 5) after two weeks of acclimatization as follows: (1) N2 (25 mg/kg); (2) ABX (MedChemExpress, Monmouth Junction, NJ, USA) (70 mg/kg); (3) vehicle of the N2 (equal volume)—saline containing 5% dimethyl sulfoxide (DMSO; Sigma-Aldrich, St. Louis, MO, USA) and 20% Sulfobutylether-β-Cyclodextrin (SBE-β-CD; MedChemExpress, Monmouth Junction, NJ, USA); (4) vehicle of the ABX (equal volume)—saline. All test substances were administered intraperitoneally to each group of mice daily for 13 days. After 1 h of the last administration, the animals were sacrificed by cervical dislocation; their brains were removed from the skull, cut along the midsagittal plane, and the prefrontal cortex and midbrain were dissected.

#### 4.7.2. Mice with MPTP-Induced PD

Mice with a toxic model of the early symptomatic stage of PD were used to conduct stage 2 of the animal studies. To induce the model, mice were given four subcutaneous injections of MPTP (12 mg/kg) at 2-h intervals on day 7, whereas control animals received an equal volume of saline [56]. Since MPTP administration induces rapid neuronal death, a pre-treatment schedule with the studied compounds was selected to evaluate whether they could enhance neuronal resistance to degeneration. The mice were divided into 4 groups (*n* = 10) after two weeks of acclimatization as follows: (1) MPTP (Sigma-Aldrich, St. Louis, MO, USA) (4 × 12 mg/kg) and N2 (22 mg/kg on days 1–6; 11 mg/kg on days 9–14); (2) MPTP (4 × 12 mg/kg) and ABX (70 mg/kg on days 1–6; 35 mg/kg on days 9–14); (3) MPTP (4 × 12 mg/kg) and vehicle (equal volume)—saline containing 5% DMSO and 20% SBE-β-CD; (4) saline (equal volume) and vehicle (equal volume)—saline containing 5% DMSO and 20% SBE-β-CD. The test substances were administered intraperitoneally to the mice over a 14-day protocol (12 days of treatment total, excluding days 7–8). The day after the last injection, half of the animals (*n* = 5 per group) were sacrificed by cervical dislocation; the brains were removed from the skull, cut along the midsagittal plane, and the prefrontal cortex and striatum were dissected. The remaining animals (*n* = 5 per group) underwent transcardial perfusion as described below.

### 4.8. GCase Activity and Lysosphingolipid Level

GCase activity and HexSph level were measured in triplicate in PBMC-derived macrophages spots (cell concentration 2 × 10^6^ cell/mL), with each line of DA neurons derived from patient iPSCs and homogenates of the mouse brain tissues using high-performance liquid chromatography coupled with tandem mass spectrometry (HPLC-MS/MS) as described previously [43,44]. GlcSph level was not analyzed separately for technical reasons. HexSph is a mixture of two isomers, GalSph and GlcSph, detected chromatographically as a single peak [98]. Cell suspension aliquots were loaded on the filter cards (20 μL) (Whatman 903 (Cytiva, Dassel, Germany)). The iPSC-derived DA neurons were lyophilized, the mouse brain tissues were subjected to ultrasound homogenization, and then all of them were resuspended in saline. The enzyme activity GCase and HexSph level were normalized to a total protein concentration.

### 4.9. GCase Protein Level

GCase protein level was assessed using western blot. Cells were lysed with a total protein extraction kit (Millipore, Burlington, MA, USA). Total protein concentration was assessed by a Pierce BSA Protein Assay Kit (Thermo Fisher Scientific, Waltham, MA, USA). Total protein (20 µg for PBMC-derived macrophages and 30 µg for iPSC-derived DA neurons) was separated in a 7.5% Mini-PROTEAN TGX Stain-Free gel (Bio-Rad Laboratories, Hercules, CA, USA) with Tris/Glycine/SDS running buffer (Bio-Rad Laboratories, Hercules, CA, USA) and transferred to a PVDF membrane (Bio-Rad Laboratories, Hercules, CA, USA), as previously described [43]. The specific antibodies used for GCase and GAPDH are listed in the Supplementary Table S1. GCase level was normalized to GAPDH. Digital images were obtained by the chemiluminescence system ChemiDoc XRS+ (Bio-Rad Laboratories, Hercules, CA, USA) and quantified using ImageJ software (version 1.54p; National Institutes of Health, Bethesda, MD, USA).

### 4.10. Immunofluorescence Staining of GCase and Lysosomal Marker LAMP2 in PBMC-Derived Macrophages

Cultured PBMC-derived macrophages were fixed in 4% paraformaldehyde (Merck, Darmstadt, Germany) at room temperature for 15 min, permeabilized with 0.1% Triton X-100 (Sigma-Aldrich, St. Louis, MO, USA) in phosphate-buffered saline (PBS; Biolot, Saint Petersburg, Russia), and blocked in 1% bovine serum albumin (Biolot, Saint Petersburg, Russia) for 30 min. Cells were incubated with primary antibodies specific to GCase and LAMP2 for 60 min, followed by incubation with secondary antibodies at room temperature in the dark for 60 min. Samples were mounted in Fluoroshield mounting medium with DAPI (Abcam, Cambridge, UK) and visualized under a Leica confocal microscope (TCS SP5 SMD FLCS; Leica Microsystems, Wetzlar, Germany) equipped with a 63× objective at a 488 nm argon laser line, a 543 nm HeNe laser line, and a 405 nm ultraviolet laser line. The antibodies used in the work are listed in Supplementary Table S1. Images were analyzed by ImageJ software using the colocalization plugin [99]. The colocalization coefficient was determined using Pearson’s algorithm.

### 4.11. Dopamine Level

Dopamine level was determined in mouse brain tissue according to a previously published protocol [100] with slight modifications, as described previously [84]. Briefly, striatal tissues from MPTP-induced PD mice were homogenized and extracted with 0.1 M perchloric acid, with 250 pmol/mL of 3,4-dihydroxybenzylamine added as an internal standard. After centrifugation (10,000× *g* for 15 min at 4 °C), dopamine was measured in the supernatant by ion-pair high-performance liquid chromatography (HPLC) with electrochemical detection using a reversed-phase Nucleodur C18 Gravity column (4.6 × 250 mm, 5 μm particle size; Macherey-Nagel, Düren, Germany) on a System Gold liquid chromatograph (Beckman Coulter, Brea, CA, USA) equipped with a System Gold 125 pump, Rheodyne 7125 injector, and a 20 μL sample loop. The mobile phase was 0.1 M citrate-phosphate buffer (pH 3.0) containing 1.1 mM octane sulfonic acid, 0.1 mM EDTA, and 9% acetonitrile at a flow rate of 1 mL/min. Detection was performed using an EC3000 electrochemical detector (RECIPE Chemicals + Instruments, Munich, Germany) with a ClinLab ECD cell (Model Sputnik), a glassy carbon working electrode (+0.85 V), and an Ag/AgCl reference electrode. Chromatograms were recorded and processed using Multichrom software (version 1.5; Ampersand, Moscow, Russia).

### 4.12. Immunohistochemical Staining of Tyrosine Hydroxylase and NeuN in Brain Tissues

#### 4.12.1. Whole Animal Brain Perfusion

Mice from the second experiment (*n* = 5 per group) were anesthetized by inhalation of 2.5% isoflurane (Laboratorios Karizoo, Barcelona, Spain) at 0.8 L/min until loss of pedal withdrawal reflex and surgical anesthesia were confirmed. Transcardial perfusion was performed according to the protocol in [101]. Briefly, after the animal’s cardiovascular system was connected to the perfusion apparatus, mice were perfused with 1X PBS at 80 mm Hg until the liver was cleared of blood. The perfusion medium was then changed to 4% paraformaldehyde in 0.1 M PBS, with the pressure gradually increased to 130 mm Hg and held constant. Upon observation of fixation tremors and pronounced rigidity, animals were decapitated, and brains were harvested following craniotomy.

#### 4.12.2. Tissue Preparation and Cryosectioning

The whole brain was placed in individual tubes containing at least 20 volumes of the same fixative solution and incubated overnight at 4 °C on a rotator. The following day, the post-fixed samples were rinsed 3 times in 1X PBS, blotted dry on filter paper for 5 min, transferred to fresh tubes filled with 70% ethanol, and incubated on a rotator for 2 h. Subsequently, the samples were serially incubated in 80%, 96%, and again 96% ethanol for 40 min each on a rotator, followed by a final dehydration in absolute ethanol for 40 min, blotted dry on filter paper for 5 min. Tissue samples were then incubated in 30% sucrose (Himmed, Moscow, Russia) in 1X PBS at 4 °C overnight until they sank to the bottom of the vial. To isolate the SN, each brain was coronally divided into three blocks using a 0.5 mm brain matrix; the position of the SN was determined according to the Mouse Brain Atlas [57]. Cryoprotected samples were embedded into the Tissue-Tek O.C.T. Compound (Sakura Finetek, Torrance, CA, USA) on dry ice until fully frozen and stored at −80 °C overnight to ensure complete solidification. Finally, the middle portion of the brain samples containing complete SN were serially sectioned at 40 µm thickness using an RWD Minux FS800 cryostat (RWD Life Science, Shenzhen, China), yielding about 36 cryosections per sample that encompassed the entire SN.

#### 4.12.3. Immunofluorescent Staining of Mouse Brain Cryosections

Immunofluorescence staining was performed on free-floating cryosections containing the entire SN to detect DA neuron markers (TH and the neuronal nuclear marker NeuN), as previously described [84]. The antibodies used are listed in Supplementary Table S1.

#### 4.12.4. Image Acquisition and Analysis

Cryosections containing complete SN were visualized under a Leica confocal microscope equipped with a 63× objective at a 488 nm argon laser line, a 633 nm HeNe laser line, and a 405 nm ultraviolet laser line. The specimens were optically sectioned, yielding 30 Z-planes per section, with a 0.67 µm interval. The set of sections per brain sample consisted of sections at Bregma −3.2 mm, −3.3 mm, −3.4 mm [102]. The images were analyzed with the ImageJ software using its object counter capabilities, as described in [103]. Initial image processing included filtering with the Gaussian blur (σ = 2.0), conversion to 8-bit images, and image binarization with the Otsu thresholding method. The “Despeckle”, “Fill holes”, and “Close” binary operations were used to reduce the noise and cover the blank space occupied by the cell nuclei. The widest portions of five TH^+^ neurons were identified to measure the area of the neurons. The minimal and maximal values were used for size filtering with the 3D object counter. The same rules were applied to the 3D cell segmentation and counting of NeuN-positive cells and DAPI-stained nuclei.

### 4.13. Statistical Analysis

Statistical analyses and data visualization were performed using IBM SPSS Statistics (version 21.0; IBM Corp., Armonk, NY, USA) and GraphPad Prism (version 10.0; GraphPad Software, San Diego, CA, USA). All continuous variables were first tested for normality using the Shapiro–Wilk test. For normally distributed data, statistical significance was determined using a paired *t*-test or a one-way ANOVA. For non-normally distributed data, comparisons between groups were performed using the Mann–Whitney *U*-test or Wilcoxon signed-rank test. Clinical data are presented as mean ± standard error of the mean. Experimental data are presented as mean ± standard error of the mean for normally distributed data, and as median (min–max) for non-normally distributed data. The level of significance was set at *p* < 0.05.

## 5. Conclusions

In summary, several strategies today are developed to treat human diseases associated with GCase deficiency in the brain, including the fusion enzymes penetrating the blood–brain barrier [104,105], gene therapy and pharmacological chaperones. Among these approaches, activators of GCase might offer a benefit for GBA1-PD treatment compared to wild-type enzyme delivery strategies, particularly since ER stress is considered a key driver of GBA1-PD pathogenesis [29,60,106]. Here, we reported the novel allosteric GCase activator N2, demonstrated its ability to bind GCase in in silico studies and enhance GCase translocation to the lysosomes on patient-derived cells derived from GD and GBA1-PD patients. Eventually, we demonstrated its neuroprotective properties in mice with MPTP-induced parkinsonism.

Our study strongly supports the efficiency of allosteric GCase activators as neuroprotective drugs. Further studies are needed to translate our preclinical result into clinical trials, to assess the potential synergistic effect with other treatments and to evaluate the potency of allosteric GCase activators to exacerbate neuroprotective effects in a broader range of neurodegenerative disorders.

## 6. Patents

Patent RU 2 809 824 C1. G.V. Baydakova; V.M. Boitsov; A.K. Emelyanov; E.Yu. Zakharova; F.M. Ibatullin; A.D. Izyumchenko; A.E. Kopytova; I.V. Miliukhina; M.A. Nikolaev; S.N. Pchelina; G.N. Rychkov; A.A. Cheblokov. Allosteric pharmacological chaperone restoring glucocerebrosidase activity. 19 December 2023.

## Figures and Tables

**Table 1:** Binding free energies (ΔGbind, kcal/mol) calculated by MM/GBSA for the five docked poses of compound N2 in complex with GCase p.N370S during 50 ns MD simulations.

Pose ID	ΔGbind, kcal/mol	Complex Stability
p3	−20.75 ± 4.58	Unstable
p4	−38.01 ± 3.49	Unstable
p5	−17.43 ± 3.83	Unstable
**p10**	**−46.69 ± 7.95**	**Stable**
p15	−31.09 ± 4.60	Stable

**Table 2:** Demographics and clinical data of the GD, GBA1-PD patients and controls.

Characteristic	GD	GBA1-PD	Controls
***n***	10	6	7
**Age, years**	39.5 ± 3.7	55.5 ± 3.5	41.3 ± 4.2
**Male, %**	50	17	42
***GBA1* pathogenic variant (*n*)**	10 p.N370S/p.L444P (4) p.N370S/p.R120W (2) p.N370S/ p.Leu29Alafs*18 (1) p.N370S/- (1) p.N370S/p.W184R (1) p.N370S/p.V375L (1)	6 p.N370S/N (4) p.L444P/N (2)	NA

## Data Availability

Characterization of iPSCs are presented in the Human Pluripotent Stem Cell Registry (hPSCreg): for GBA1-PD (https://hpscreg.eu/cell-line/ICGi034-A; https://hpscreg.eu/cell-line/ICGi034-B; https://hpscreg.eu/cell-line/ICGi034-C) and for healthy individuals (https://hpscreg.eu/cell-line/ICGi021-A; https://hpscreg.eu/cell-lineICGi022-A; https://hpscreg.eu/cell-line/ICGi057-A) all accessed on 20 September 2026.

## Supplementary Materials

The following supporting information can be downloaded at: https://www.mdpi.com/article/10.3390/ijms27219602/s1.

## Abbreviations

6-OHDA	6-hydroxydopamine
ABX	ambroxol
DA	dopaminergic
GBA1-PD	Parkinson’s disease associated with *GBA1* pathogenic variants
GCase	β-glucocerebrosidase
GD	Gaucher disease
GalSph	galactosylsphingosine
GlcCer	glucosylceramide
GlcSph	glucosylsphingosine
HexSph	hexosylsphingosine
HPLC	high-performance liquid chromatography
HPLC-MS/MS	high-performance liquid chromatography coupled with tandem mass spectrometry
iPD	idiopathic Parkinson’s disease
iPSC	induced pluripotent stem cell
MD	molecular dynamics
MPTP	1-methyl-4-phenyl-1,2,3,6-tetrahydropyridine
NCGC607	NCGC00241607
NCGC758	NCGC00188758
PBMC	peripheral blood mononuclear cell
PCs	pharmacological chaperones
PD	Parkinson’s disease
SN	substantia nigra
TH	tyrosine hydroxylase
