# Supplementary material for: A Novel Glucocerebrosidase Allosteric Activator Prevents MPTP-Induced Neurodegeneration in Mice
Source: Int J Mol Sci. 2026 Oct 27;27(21):9602. doi: 10.3390/ijms27219602 (PMC13762957; doi:10.3390/ijms27219602)
Supplement: Supplementary file 1 [file ijms-27-09602-s001.zip › ijms-4608155-supplementary.pdf]

Supplementary materials

Chemical synthesis of 2-(2-(2-((4-Iodophenyl)amino)-2-oxoethoxy)benzoyl)-*N*-phenylhydrazinecarboxamide (7 (N2))

General Information: Nuclear magnetic resonance spectra (^{1}H (400 MHz) and ^{13}C (100 MHz)) were recorded on a Bruker Avance 400 spectrometer in DMSO-d_6 solution. ^{13}C NMR spectra were registered with broad-band proton decoupling. Chemical shifts (δ) are reported relative to residual undeuterated solvent**.** The signal patterns are indicated as follows: s (single), d (doublet), t (triplet). Integrals are given in accordance with assignments, coupling constants are reported in Hz. High resolution mass spectra were recorded on a HRMS-ESI-QTOF mass-analyzer, electrospray ionization, positive mode. The progress of reaction was monitored by TLC on Silufol UV-254 plates using UV light for detection. All solvents were dried by standard procedures and freshly distilled prior to use. All reagents used were purchased from commercial suppliers and were used without further purification.

2-Chloro-*N*-(4-iodophenyl)acetamide (3). Chloroacetyl chloride (**1**) (7.54 mL, 96 mmol) was added dropwise to a stirred, cooled (0°C) solution of 4-iodoaniline (**2**) (17.52 g, 80 mmol) and Et_3N (14.5 mL, 104 mmol) in anhydrous dichloromethane (160 mL). The reaction mixture was stirred for 4 hours at room temperature, washed with cold water (2 × 100 mL), dried over anhydrous Na_2SO_4, and the solvent was evaporated under reduced pressure. The yield of 2-chloro-N-(4-iodophenyl)acetamide (**3**), which was sufficiently pure for the next experiment, was 76% (18.2 g, white solid).

2-(2-Hydroxybenzoyl)-*N*-phenylhydrazinecarboxamide (6). Freshly distilled phenyl isocyanate (**5**) (10.9 mL, 100 mmol) was added dropwise over 15–20 minutes to a stirred, cooled (0°C) solution of salicyloylhydrazide (**4**) (15.22 g, 100 mmol) in anhydrous DMF (50 mL). The reaction mixture was stirred for 6 hours at room temperature under an argon atmosphere. The resulting precipitate was filtered off, washed with isopropyl alcohol, and air-dried. This yielded 12.8 g (47%) of hydrazide **6** as a colorless solid. An additional amount of product **6** could be obtained by adding 20 mL of water to the mother liquor (heated to 80°C), followed by filtering the resulting precipitate and washing it with isopropyl alcohol. The total yield of 2-(2-hydroxybenzoyl)-N-phenylhydrazinecarboxamide (**6**) was 17.2 g (63%).

2-(2-(2-((4-Iodophenyl)amino)-2-oxoethoxy)benzoyl)-*N*-phenylhydrazinecarboxamide (7). To a mixture of 2-chloro-N-(4-iodophenyl)acetamide (**3**) (10 g, 33.8 mmol), 2-(2-hydroxybenzoyl)-*N*-phenylhydrazinecarboxamide (**6**) (9.16 g, 33.8 mmol), and KI (280.5 mg, 1.7 mmol) in dry dimethylformamide (270 mL), finely ground calcined potassium carbonate (4.66 g, 33.8 mmol) was added, and the resulting suspension was stirred for 24 hours at room temperature under an argon atmosphere. Then, an equal volume of water was added to the reaction mixture; the resulting precipitate was filtered off and washed with water and isopropyl alcohol. The obtained crude product **7 (N2)** was recrystallized from ethanol. The yield of white solid **7 (N2)** was 9.1 g (51%).

^{1}H NMR (400 MHz, DMSO-d_6): δ = 4.90 (s, 2H), 6.98 (t, 1H, 7 Hz), 7.14 (t, 1H, 7 Hz), 7.22 (d, 1H, 8 Hz), 7.25 (t, 2H, 8Hz), 7.45 (d, 2H, 8Hz), 7.48 (d, 2H, 9 Hz), 7.54 (d, 1H, 9 Hz), 7.58 (d, 2H, 9 Hz), 7.75 (d, 1H, 8 Hz), 8.31 (s, 1H), 8.85 (s, 1H), 10.28 (s, 1H), 10.30 (s, 1H). ^{13}C NMR (100 MHz, DMSO-d_6): δ = 167.2, 166.6, 158.0, 140.0, 138.3, 137.8, 133.3, 130.8, 129.1, 122.9, 122.5, 122.1, 119.0, 116.2, 114.5, 88.5, 88.02, 68.4. HRMS (ESI): *m/z* $[M + Na]^+$ calculated for $C_{22}H_{19}IN_4O_4Na^+$: 553.0349, found: 553.0339.

Table S1. List of used antibodies

Experiment	Antibody	Manufacturer	Cat. No.	Host/Type	Dilution/Concentration
	**Primary antibodies**				
ICC/IF	Anti-TH	Millipore, Burlington, VT, USA	AB152	Rabbit polyclonal IgG	1:400
ICC/IF	Anti-TUBB3	Covance, Princeton, NJ, USA	MMS-435P	Mouse monoclonal IgG2a	1:1000
WB	Anti-GBA	Abcam, Cambridge, UK	ab125065	Rabbit monoclonal IgG	1:500
WB	Anti-GAPDH	Sigma-Aldrich, Darmstadt, Germany	SAB2108266	Rabbit polyclonal IgG	1:18000
ICC/IF	Anti-GBA	Sigma-Aldrich, Darmstadt, Germany	WH0002629M1	Mouse monoclonal IgG	7 μg/mL
ICC/IF	Anti-LAMP2	Sigma-Aldrich, Darmstadt, Germany	SAB2701987	Rabbit polyclonal IgG	1:400
IHC	AffiniPure Fab Fragment Goat Anti-Mouse IgG (H+L)	Jackson ImmunoResearch, West Grove, PA, USA	115-007-003	Goat	40 μg/ml
IHC	Anti-TH	Servicebio, Wuhan, China	GB15182-100	Rabbit monoclonal IgG	1:300
IHC	Anti-NeuN	Servicebio, Wuhan, China	GB15138-100	Mouse monoclonal IgG	1:300
	**Secondary antibodies**				
ICC/IF	Alexa Fluor 488 goat anti-rabbit	Thermo Fisher Scientific, Waltham, MA, USA	A11008	Goat	1:400

	IgG (H+L)				
ICC/IF	Alexa Fluor 568 goat anti-rabbit IgG (H+L)	Thermo Fisher Scientific, Waltham, MA, USA	A11011	Goat	1:400
ICC/IF	Alexa Fluor 488 goat anti-mouse IgG2a	Thermo Fisher Scientific, Waltham, MA, USA	A21131	Goat	1:400
WB	Goat anti-rabbit IgG H&L (HRP)	Abcam, Cambridge, UK	ab6721	Goat	1:5000
ICC/IF	Cy3 sheep anti-mouse IgG (H+L)	Jackson ImmunoResearch, West Grove, PA, USA	515-165-062	Sheep	1:400
ICC/IF	Alexa Fluor 488 goat anti-rabbit	Jackson ImmunoResearch, West Grove, PA, USA	111-545-045	Goat	1:400
IHC	Cy5 goat anti-mouse IgG (H+L)	Servicebio, Wuhan, China	GB27301	Goat	1:200
IHC	AF 488-goat anti-rabbit IgG (H+L)	Servicebio, Wuhan, China	GB25303	Goat	1:200

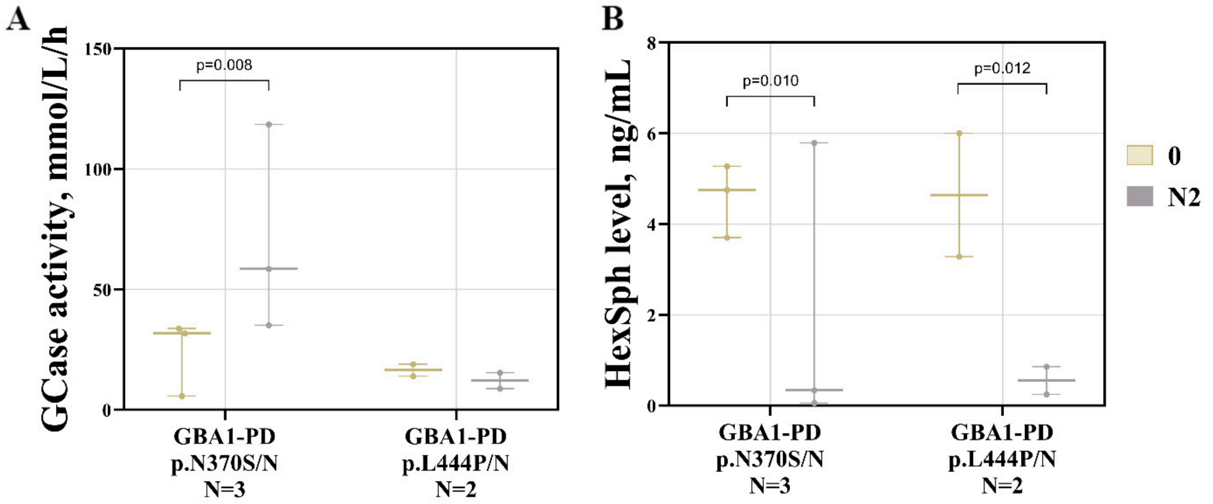

Figure S1. Genotype-dependent effects of N2 treatment on PBMC-derived macrophages from GBA1-PD patients. GCase activity (**A**) and HexSph level (**B**) in PBMC-derived macrophages from GBA1-PD patients (*GBA1* mutations: p.N370S (n = 3), p.L444P (n = 2)) after N2 (4 µM) treatment.

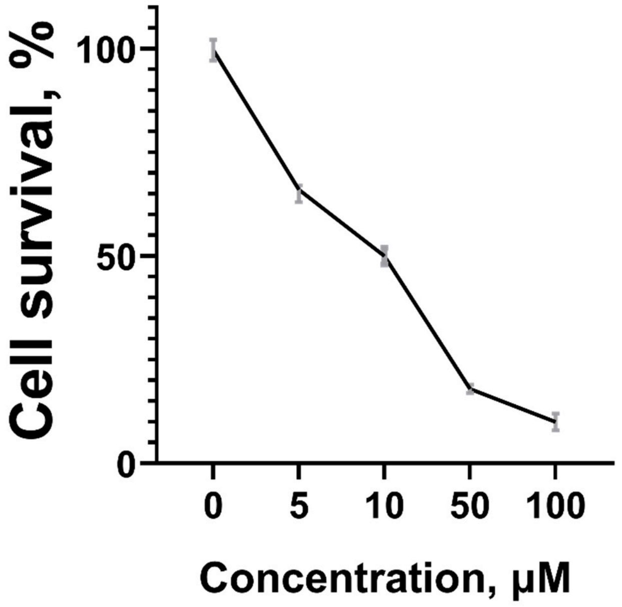

Figure S2. Definitions the optimal concentrations of N2 without noticeable toxic effects on PBMC-derived macrophages. Colorimetric method (MTS test) for determining the number of viable cells in proliferation or cytotoxicity tests depending on the concentration of N2.
